# The Phytochemical and Nutritional Composition of Shallot Species (*Allium × cornutum*, *Allium × proliferum* and *A. cepa* Aggregatum) Is Genetically and Environmentally Dependent

**DOI:** 10.3390/antiox11081547

**Published:** 2022-08-10

**Authors:** Nikola Major, Josipa Perković, Igor Palčić, Iva Bažon, Ivana Horvat, Dean Ban, Smiljana Goreta Ban

**Affiliations:** 1 Department of Agriculture and Nutrition, Institute of Agriculture and Tourism, K. Hugues 8, 52210 Poreč, Croatia; 2 Centre of Excellence for Biodiversity and Molecular Plant Breeding, Svetošimunska 1, 10000 Zagreb, Croatia

**Keywords:** shallot, flavonoid, inulin, FOS, antioxidant, mineral composition, pungency, agroclimatic conditions

## Abstract

Shallots are a perennial plant from the Alliaceae family, classified with the common onion under the name of the *Allium cepa* Aggregatum group. The term shallot is also used for diploid and triploid viviparous onions, known as *Allium × proliferum* (Moench) Schrad and *Allium × cornutum* Clementi ex Vis., respectively. In this study, we compared the dry matter, pyruvic acid content, sugar content, flavonoid content, antioxidant capacity and mineral composition of 34 shallot accessions falling into three shallot species (*Allium × cornutum*, *Allium × proliferum* and *A. cepa* Aggregatum). Shallot accessions belonging to the *A.× cornutum* and *A. × proliferum* groups are characterized by high dry matter content (around 25%), of which a little less than 50% is formed of inulin-type sugars, polysaccharides, considered an excellent prebiotic with beneficial effects on human health. On the other hand, accessions belonging to the *A. cepa* Aggregatum group have lower dry matter content and, as a result, lower pungency (measured as pyruvic acid content), making them more suitable for fresh consumption by a broader range of consumers, but, at the same time, abundant in phenolic compounds, especially quercetin and isorhamnetin glycosides. We also observed a greater biodiversity among accessions within the *A. cepa* Aggregatum group in all the analyzed physico-chemical parameters compared to the other shallot groups. The investigated shallot accessions have an excellent in vitro antioxidant capacity, as well as excellent nutritional properties.

## 1. Introduction

Alliums are widely cultivated around the world and are consumed either as vegetables or as a condiment due to their distinctive aroma and health benefits. Onions (*Allium cepa* L.) are often consumed cooked in warm dishes and even raw in salads [1]. Several studies have demonstrated that onion consumption has a very broad list of health benefits, including antimicrobial, anti-inflammatory, anticancer, anti-diabetic and cardiovascular protection properties [2,3,4,5,6].

The health benefits come from the phytochemicals present in all Alliums, including cultivated species such as garlic, leek, the common onion and shallots, as well as wild species, such as *Allium ursinum* L. These phytochemicals are organosulfur compounds, phenolic compounds, polysaccharides and saponins. Shallots are known for their high content of phenolics, especially the flavanol quercetin, in its conjugated form, with saccharides [7].

Organosulfur compounds are also responsible for the aroma and pungency of onions and can be easily measured by their pyruvic acid content, which is a byproduct of the breakdown reaction of S-alk(en)yl-L-cysteine sulfoxides into volatile and non-volatile organosulfur compounds upon tissue damage [8].

Polysaccharides are abundant in several Allium species, such as garlic and shallots, but less so in common onions [9]. The polysaccharides found in shallots are inulin-type fructo-oligosaccharides [10]. These saccharides are soluble, resistant to hydrolysis in the human gut, and can be fermented by human gut microbiota to produce short-chain fatty acids and other by-products that possess immunomodulatory effects [11,12]. These saccharides have a low antioxidant capacity compared to powerful antioxidants such as vitamin C [13], but are known to enhance the antioxidant capacity of the human plasma [14].

Shallots are a perennial plant from the *Alliaceae* family, formerly considered a separate species (*Allium ascalonicum*) but now classified within the common onion group under the name *A. cepa* Aggregatum [15].

In Croatia, the term shallot is used for diploid and triploid viviparous onions, also known as *Allium* × *proliferum* (Moench) Schrad (*A. × proliferum*) and *Allium* × *cornutum* Clementi ex Vis., respectively [16]. The *A. cepa* Aggregatum group is the most cultivated shallot species in Europe and, based on their morphological traits, can be categorized into two subgroups: one has narrow, pear-shaped bulbs (Shallot type—SH), making it distinguishable from the other, which has rounder bulbs (Potato onion type—PO) [15,17]. The categorization into these two groups seems quite simple, being based on the morphological bulb traits, but the great biodiversity of the *A. cepa* Aggregatum group is responsible for the occurrence of many intermediate types of varying bulb size, shape, and number of clusters [18].

Two main factors influence the phytochemical composition of any plant species: the genetic material [19] and the environment in which it grows [20]. More specifically, the composition of phytochemicals is regulated by environmental factors, such as temperature, available light, precipitation and humidity, which in turn entrain the plant’s circadian clock in an orchestrated effort to finely tune the phytochemicals to the specific needs or the current demands of the plant [21]. Thus, the seasonal changes in environmental conditions can greatly affect the current phytochemical status of the plant, and there is a need for studies which take this aspect into consideration.

The aim of this study is to provide a comprehensive phytochemical and nutritional characterization of shallot accessions from the ex-situ collection of the Institute of Agriculture and Tourism, Poreč, Croatia, over two harvest years, with an emphasis on the differentiation of the *A. cepa* Aggregatum SH and PO subgroups, as well as the *A. × cornutum* and *A. × proliferum* groups, using multivariate statistical analysis. We build upon our previous work [22] by increasing the number of accessions studied, including two harvest years’ worth of data, and by expanding the list of analytical methods used to map the phytochemical biodiversity of the shallot accessions. Additionally, we also build upon the work by Perković et al. [17], where shallot morphological descriptors were used to successfully discriminate between various shallot groups, including the differentiation of the *A. cepa* Aggregatum SH and PO subgroups.

## 2. Materials and Methods

### 2.1. Plant Material

In the years 2018 and 2019, 34 shallot accessions belonging to the ex situ collection of the Institute of Agriculture and Tourism were planted at the Institute’s experimental farm. The procedure was described by Perković et al. [17]. Briefly, bulbs of a standard size were chosen for each accession and planted at the beginning of October at a distance of 20 cm in rows with 30 cm between rows, and 40 bulbs in total were planted for each accession. Agricultural practices for onion growing without irrigation were applied, and the weeds were removed manually. Fertilizer NPK (5:20:30) was incorporated at 500 kg ha^−1^ before planting, and 45 kg ha^−^^1^ N was applied (urea source) at the beginning of March each year. The shallots were harvested at the begging of July, when 50% of the pseudo stems were bent over and left to cure under shade for a month.

The fresh bulbs were frozen at −80 °C, freeze-dried (Labogene Coolsafe 95-15 Pro, Allerød, Denmark) and finely milled to 0.2 mm (Retsch ZM200, Haan, Germany) prior to the extraction process.

The ultrasound-assisted extraction of sugars and phenolic compounds was performed by sonicating 75 mg of the freeze-dried tissue in 1,5 mL of 80% aqueous methanol for 30 min (MRC DCG-250H, Holon, Israel). Afterwards, the extracts were left to macerate on an orbital shaker (GFL 3005, Lab Unlimited, Dublin, Ireland) at 150 rpm at 20 °C for 3.5 h. The extracts were centrifuged at 15,000× *g* for 5 min (Domel Centric 350, Železniki, Slovenia) and the supernatants were filtered through a 0.22 µm nylon filter into an HPLC vial. The samples were stored at −80 °C until the time of analysis. The methanolic extracts were used to determine the total antioxidant capacity, total phenolic content, as well as the sugar and flavonoid profile.

The dry matter was determined by hot air drying (Memmert UF160, Schwabach, Germany) at 105 °C until a consistent weight was obtained in three replications.

### 2.2. Determination of the Shallot Sugar Profile

The analysis of the inulin, sucrose, fructose and sucrose content was carried out using an HPLC system, consisting of a solvent delivery unit (Shimadzu Nexera LC-40DX3, Kyoto, Japan), an autosampler (Shimadzu Nexera SIL-40CX3, Kyoto, Japan), a column oven (Shimadzu Nexera CTO-40C, Kyoto, Japan) and a refractive index detector (Shimadzu RID-20A, Kyoto, Japan). Chromatographic separation was achieved by injecting 10 µL of the sample into a 300 × 8 mm calcium ion exchange column of a 9 µm particle size (Dr. Maisch ReproGel Ca, Ammerbuch, Germany), held at 80 °C using deionized water as the mobile phase (1 mL/min, isocratic elution). The retention times and peak areas of the investigated sugars were compared to the analytical standards for identification and quantification. Linear calibration curves were obtained with serial dilutions of 0.25, 0.50, 1.00, 2.50, 5.00, 7.50 and 10.00 g/L of inulin (y = 1850.07x + 109.39, coefficient of determination, R^2^ = 0.9998, recovery: 100.4 ± 0.2%), sucrose (y = 2265.73x + 29.97, coefficient of determination, R^2^ = 0.9998, recovery: 99.9 ± 2.3%), glucose (y = 2224.75x + 28.33, coefficient of determination, R^2^ = 0.9999, recovery: 99.8 ± 1.8%) and fructose (y = 2233.53x + 47.37, coefficient of determination, R^2^ = 0.9998, recovery: 100.0 ± 0.6%).

### 2.3. Determination of the Shallot Flavonoid Profile

The analysis of the flavonoid profile was performed using an HPLC system consisting of two solvent delivery units (Shimadzu Nexera LC-40DX3, Kyoto, Japan), an autosampler (Shimadzu Nexera SIL-40CX3, Kyoto, Japan), a thermostated column compartment (Shimadzu Nexera CTO-40C, Kyoto, Japan) and a photo diode array detector (Shimadzu Nexera SPD-M40, Kyoto, Japan). The reversed-phase separation of the targeted compounds was achieved by injecting 5 µL of the sample extract into a C18, 2.1 mm × 150 mm, 2.7 µm core-shell column (Advanced Materials Technology, Wilmington, DE, USA), held at 37 °C, using a linear binary gradient elution of mobile phase A (water/0.1% formic acid) and mobile phase B (acetonitrile/0.1% formic acid) at 0.35 mL/minute, for 0 min to 2 min: 95%A; 2 min to 20 min: 95%A to 50%A; 20 min to 21 min: 50%A to 5%A; 21 min to 23 min: 5%A; 23 min to 24 min: 5%A to 95%A; and 24 min to 30 min: 95%A. Additionally, the identification of the target compounds was performed by comparing the retention times and characteristic parent/product ions to the analytical standards (quercetin, quercetin-3-glucoside, quercetin-4′-glucoside and quercetin-3,4′-diglucoside) using an LC-ESI-QqQ, which consisted of two solvent delivery units (Shimadzu Nexera LC-40DX3, Kyoto, Japan), an autosampler (Shimadzu Nexera SIL-40CX3, Kyoto Japan), a thermostated column compartment (Shimadzu Nexera CTO-40C, Kyoto, Japan) and a QqQ mass spectrometer (Shimadzu LCMS8045, Kyoto, Japan). The reversed-phase separation was performed by injecting 1 µL of the sample extract into a C18, 2.1 mm × 150 mm, 2.7 µm core-shell column (Advanced Materials Technology, Wilmington, DE, USA), held at 37 °C, using a linear binary gradient elution of mobile phase A (water/0.1% formic acid) and mobile phase B (acetonitrile/0.1% formic acid) at 0.35 mL/minute, for 0 min to 0.75 min: 98%A; 0.75 min to 15 min: 98%A to 50%A; 15 min to 15.1 min: 50%A to 0%A; 15.1 min to 20 min: 0%A; 20 min to 20.1 min: 0%A to 98%A; and 20.1 min to 25 min: 98%A. Quercetin-7,4′-diglucoside and isorhamnetin-4′-glucoside were tentatively identified by LC-ESI-QqQ, using the characteristic parent/product ions obtained by the fragmentation of the quercetin-3,4′-glucoside and isorhamnetin-3-glucoside analytical standards, respectively, while isorhamnetin-3,4′-diglucoside was identified using published parent/product ions from the literature sources [23,24]. The compounds were quantified at 360 nm against calibration curves obtained with 1, 5, 10, 25, 50, 75 and 100 mg/L serial dilutions of the quercetin (y = 1.5997x − 0.12402, coefficient of determination, R^2^ = 0.9994, recovery: 100.3 ± 1.3%), quercetin-3-glucoside (y = 1.58060x − 0.03397, coefficient of determination, R^2^ = 0.9999, recovery: 98.7 ± 0.2%), quercetin-4′-glucoside (y = 1.09028x − 3.63246, coefficient of determination, R^2^ = 0.9994, recovery: 99.2 ± 0.8%) and quercetin-3,4′-diglucoside (y = 1.78920x − 0.41645, coefficient of determination, R^2^ = 0.9988, recovery: 99.3 ± 0.3%) standards. Quercetin-7,4′-diglucoside and isorhamnetin-3,4′-diglucoside were quantified using the calibration curve of quercetin-3,4′-diglucoside, while isorhamnetin-4′-glucoside was quantified using the calibration curve of isorhamnetin-3-glucoside (y = 0.4792x − 0.0581; serial dilutions of 1.0, 2.5, 5.0, 7.5 and 10.0 mg/L; coefficient of determination, R^2^ = 0.9999).

### 2.4. Total Phenolic Content and Total Antioxidant Capacity

The total phenolic content (TPC) was evaluated using the Folin-Ciocalteu assay [25]. Briefly, 20µL of the sample was mixed with 140 µL of 0.2 M Folin-Ciocalteu reagent, and, after 1 min, 140 µL of 6% sodium carbonate was added. The reaction mixture was incubated at 25 °C for 60 min and the absorbance was read at 750 nm (Tecan Infinite 200 Pro M Nano+, Männedorf, Switzerland). The TPC was standardized against the gallic acid and expressed as the mg of gallic acid equivalents per g sample in DW. The results were calculated against a standard curve of gallic acid (y = 3.7867x − 0.2144; serial dilutions of gallic acid: 12.5, 25, 50, 75, 100, 150 and 250 mg/L; coefficient of determination, R^2^ = 0.9999, recovery: 102.0 ± 2.9%) and expressed as mg GAEQ/g DW.

The total antioxidant activity was evaluated using the FRAP assay [26] and the DPPH radical scavenging activity assay [27]. Briefly, 100 µL of the sample was mixed with 200 µL of either freshly prepared FRAP reagent or 0.02 M DPPH radical for the FRAP or DPPH assays, respectively. The antioxidant capacity using the FRAP assay was evaluated after 10 min of the reaction time, at 25 °C, by reading the absorbance at 593 nm (Tecan Infinite 200 Pro M Nano+, Männedorf, Switzerland), while the DPPH radical scavenging capacity was evaluated after 30 min of the reaction time, at 25 °C, by reading the absorbance at 517 nm (Tecan Infinite 200 Pro M Nano+, Männedorf, Switzerland).

FRAP values were calculated against an Fe^2+^ calibration curve (y = 0.0168x − 0.002; serial dilutions of Fe^2+^: 20, 40, 80, 120, 160, 200 and 250 µM; coefficient of determination, R^2^ = 0.9999, recovery: 101.8 ± 1.6%) and expressed as µmol Fe^2+^/g DW. DPPH radical scavenging ability values were calculated against a standard curve of Trolox (y = −0.0137x + 0.0133; serial dilutions of Trolox—2, 5, 10, 25, 50, 75 and 100 µM; coefficient of determination, R^2^ = 0.9997, recovery: 103.7 ± 1.2%) and expressed as µmol TEQ/g DW, respectively.

### 2.5. Pyruvic Acid Content

Pyruvic acid was extracted by macerating 75 mg of freeze-dried plant tissue in 1.5 mL of deionized water at 25 °C for 30 min. The extract was centrifuged at 15,000× *g* for 5 min and the supernatant was filtered through a 0.22 µm nylon filter into an HPLC vial. The samples were analyzed immediately after extraction. The pyruvic acid content was determined by HPLC, consisting of a solvent delivery unit (Varian 230, Palo Alto, CA, USA), an autosampler (Varian 410, Palo alto, CA, USA) and a UV/Vis detector (Varian 325, Palo Alto, CA, USA). The chromatographic separation was achieved by injecting 10 µL of the sample into an aqueous C18, 4.6 mm × 250 mm column of a 3 µm particle size (Bischoff Analysentechnik, Leonberg, Germany), held at 35 °C, with the isocratic elution of the mobile phase (25 mM phosphate buffer, pH 2.5) at 0.7 mL/minute. The pyruvic acid was identified and quantified against its analytical standard with a linear calibration curve, obtained with serial dilutions of 0.20, 0.40, 0.60, 0.80 and 1.00 g/L of pyruvic acid (y = 0.07782x − 0.58921, coefficient of determination, R^2^ = 0.9994, recovery: 100.1 ± 0.7%).

### 2.6. Determination of the Shallot Mineral Composition

The determination of the macro- and micro-elements (calcium—Ca, potassium—K, phosphorus—P, sulfur—S, magnesium—Mg, aluminum—Al, boron—B, copper—Cu, iron—Fe, lithium—Li, manganese—Mn, molybdenum—Mo, sodium—Na, and zinc—Zn) was carried out with inductively coupled plasma–optical emission spectroscopy (ICP-OES), with both axial and radial viewing (ICPE-9800 Shimadzu, Kyoto, Japan) after microwave-assisted digestion (Ethos Up, Milestone, Sorisole, Italy). Briefly, 200 mg of the freeze-dried sample was digested with 6 mL of concentrated HNO_3_ and 2 mL of 30% H_2_O_2_, transferred to a polyethylene 25 mL volumetric flask and filled to the mark with ultrapure water. The samples were stored at 4 °C until the time of analysis. The method accuracy evaluation was carried out using four certified reference materials from the WEPAL dried plant material program (WEPAL, Wageningen, The Netherlands). The operating parameters were as follows: 1.20 kW of RF power, 10.0 L min^−1^ of plasma flow rate, 0.6 L min^−1^ of auxiliary gas flow rate and 0.7 L min^−1^ of carrier flow rate. The sample solutions were introduced into the plasma using a nebulizer and a cyclonic spray chamber. Argon (99.999% purity, Messer, Zaprešić, Croatia) was used to form the plasma. Elemental analytical lines were determined and quantitation was achieved by plotting linear calibration curves with spectral and background corrections of single element standards (Inorganic Ventures, Christianburg, Virginia, USA), using serial dilutions of Ca (1 mg/L to 100 mg/L), K (1 mg/L to 100 mg/L), P (1 mg/L to 100 mg/L), S (0.1 mg/L to 5 mg/L), Mg (1 mg/L to 100 mg/L), Al (0.1 mg/L to 5 mg/L), B (0.1 mg/L to 5 mg/L), Cu (0.1 mg/L to 5 mg/L), Fe (0.1 mg/L to 5 mg/L), Li (0.1 mg/L to 5 mg/L), Mn (0.1 mg/L to 5 mg/L), Mo (0.1 mg/L to 5 mg/L), Na (1 mg/L to 100 mg/L) and Zn (0.1 mg/L to 5 mg/L).

### 2.7. Statistical Analysis

All analyses conducted in this study were performed in three biological repetitions. The obtained data were analyzed by analysis of variance (ANOVA) and Partial Least Square Discriminant Analysis (PLS-DA) using Statistica 13.4 (Tibco, Inc, Palo Alto, CA, USA). Significant differences were determined at a value of *p* ≤ 0.05, and the homogenous group means were compared using Tukey’s post hoc test.

The main application of PLS regression is the prediction of dependent variables based on information on the independent variables, while reducing the dimensionality of the dataset in the form of new components [28]. Unlike unsupervised multivariate methods, such as Principal Component Analysis, PLS also uses information from the dependent variables in the formation of new components [28,29]. In the case of a PLS-DA model, a regression is formed between a set Y of binary variables, describing the categories of a categorical variable on a set X of predictor variables, and this method is especially suitable for dealing with a much larger number of predictors than observations, and with multicollinearity [30]. In this study, the PLS-DA model was used as an exploratory tool to discriminate among the studied shallot species based on the analyses performed.

## 3. Results

### 3.1. Dry Matter, Sugar Profile and Pyruvic Acid Content in Shallot Accessions

Significant effects of the harvest year, the species and their interactions were observed in the content of dry matter, pyruvic acid, inulin, sucrose, glucose and fructose in the shallot bulbs (Table 1). The highest dry matter content was observed in the *A. × cornutum* species in 2018, while in 2019 this species had a comparable dry matter content to the *A. × proliferum* species from both harvest years. The PO and SH types of the *A. cepa* Aggregatum group had comparable dry matter contents in the harvest year 2018, while in the harvest 2019 the dry matter was significantly lower in both subgroups.

All accessions belonging to the *A. × cornutum* and *A. × proliferum* species had comparable dry matter content, ranging from 26.4% in IPT211 to 24.5% in IPT245. Accessions from the *A. cepa* Aggregatum group had significantly lower dry matter content and ranged from 18.2% in IPT217 to 12.1% in IPT241.

Overall, the *A. cepa* Aggregatum PO type had significantly lower pyruvic acid content compared to the other species, albeit that this depended on the harvest year and the specific accession. The pyruvic acid content was significantly higher in the harvest year 2019 (27.6, 22.7 and 33.1 mmol/kg DW for *A. × cornutum*, *A. cepa* Aggregatum PO and *A. cepa* Aggregatum SH, respectively) compared to the harvest year 2018 (16.6, 18.1 and 14.8 mmol/kg DW for *A. × cornutum*, *A. cepa* Aggregatum PO and *A. cepa* Aggregatum SH, respectively) in all species, except in *A. × proliferum*, where a higher pyruvic acid content was observed in 2018 (35.5 mmol/kg DW) compared to 2019 (19.2 mmol/kg DW).

The highest pyruvic acid content was observed in accession IPT021, belonging to the *A. × cornutum* species (39.5 mmol/kg DW), followed by accession IPT210 from the *A. × proliferum* species (33 mmol/kg DW), and the lowest content was observed in accession IPT243 (16.4 mmol/kg DW), belonging to the *A. cepa* Aggregatum PO type.

A higher inulin content was observed in the *A. × cornutum* and *A. × proliferum* in 2019 (54.3 and 54.5 g/100 g DW, respectively), with lower values in 2018 (40.7 and 35.2 g/100 g DW, respectively), compared to the *A. cepa* Aggregatum SH and PO types. On the other hand, the inulin content was comparable between the harvest years for the *A. cepa* Aggregatum PO type (29.3 and 29.9 g/100 g DW in the years 2018 and 2019, respectively). The lowest inulin content was recorded in the *A. cepa* Aggregatum SH type from the harvest year 2019 (18.7 g/100 g DW), with higher inulin values in the harvest year 2018 (26.4 g/100 g DW).

The inulin content was divided into three groups among all the investigated accessions, where the high-range group was represented by accessions IPT023, IPT214, IPT021, IPT212, IPT213, IPT210, IPT022, IPT215 and IPT211 (ranging from 42.4 g/100 g DW in IPT023 to 51.1 g/100 g DW in IPT211), belonging to the *A. × cornutum* and *A. × proliferum species*. Accessions IPT241, IPT230, IPT245, IPT242, IPT239, IPT240, IPT228, IPT237, IPT238, IPT231, IPT243, IPT234, IPT233, IPT244, IPT232, IPT236 and IPT229 represented the lower range group of accessions, where the inulin content ranged from 17.1 g/100 g DW in IPT241 to 30.0 g/100 g DW in IPT229, belonging to the *A. cepa* Aggregatum SH and PO types, respectively. The mid-range group of accessions was represented by IPT235, IPT176, IPT226, IPT225, IPT216, IPT217, IPT218 and IPT208 from the *A. cepa* Aggregatum PO type, where the inulin content ranged from 30.1 g/100 g DW in IPT235 to 39.2 g/100 g DW in IPT208.

Significant differences were observed in the sucrose content between harvest years, where higher values were observed in the harvest year 2019 compared to the harvest year 2018 in all investigated species and types. In both growing years, a significantly higher sucrose content was found in the *A. cepa* Aggregatum PO (5.05 and 1.90 g/100 g DW in 2019 and 2018, respectively) and SH (4.16 and 1.89 g/100 g DW in 2019 and 2018, respectively) types compared to the *A. × cornutum* (0.79 and 0.38 g/100 g DW in 2019 and 2018, respectively) and *A. × proliferum* (3.02 and 1.12 g/100 g DW in 2019 and 2018, respectively). Among individual accessions, the highest sucrose content was observed in IPT225 (5.68 g/100 g DW) and the lowest in IPT215 (1.49 g/100 g DW).

The *A. cepa* Aggregatum PO and SH types from the harvest year 2019 had significantly higher glucose and fructose contents compared to the same types from the harvest year 2018 and the *A. × cornutum* and the *A. × proliferum* groups from both harvest years (except for the glucose content in *A.× proliferum* from the harvest year 2019). Both glucose and fructose were found to be most abundant in accession IPT230, while the least amount of glucose and fructose was observed in accessions IPT215 and IPT211, respectively.

### 3.2. Phenolic Compound Content and Antioxidant Activity

Phenolic compound content and antioxidant activity were affected by the growing year (except quercetin-3,4′-diglucoside, quercetin-3-glucoside, and sum of flavonoids), accessions and their interaction (Table 2). The quercetin-7,4′-diglucoside content was comparable between the *A. × proliferum* from both harvest years, the *A. × cornutum* and *A. cepa* Aggregatum SH type, both from the harvest year 2019, with the *A. cepa* Aggregatum SH from the harvest year 2018 and PO from the harvest year 2019 being very close. The least amount of quercetin-7,4′-diglucoside was detected in *A. × cornutum* and *A. cepa* Aggregatum PO, both from the harvest year 2018. The highest quercetin-7,4′-diglucoside content was observed in IPT023 and the lowest in IPT243.

Quercetin-3,4′-diglucoside was the second most abundant flavonoid in the investigated shallot accessions. In the harvest year 2018, the *A. cepa* Aggregatum SH type had the highest quercetin-3,4′-diglucoside content, followed by the *A. × proliferum* and *A. × cornutum* species, while *A. cepa* Aggregatum PO had the lowest one. In the harvest year 2019, the *A. × cornutum* had the highest quercetin-3,4′-diglucoside content, while the accessions belonging to the other species and types were significantly lower.

Regardless of the harvest year, the highest quercetin-3,4′-diglucoside content was observed in accessions IPT023 from the *A. × proliferum*, IPT021, IPT022 and IPT215 from the *A.* × *cornutum*, and IPT241 from the *A. cepa* Aggregatum SH type, ranging from 119.9 ± 17.4 mg/100 g DW in IPT023 to 143.2 ± 17.5 mg/100 g DW in IPT241.

The isorhamnetin 3,4′-diglucoside content was found to be higher in the harvest year 2019 in the *A. × cornutum* and *A. cepa* Aggregatum PO type compared to the harvest year 2018, while its content did not differ between harvest years in the *A. cepa* Aggregatum SH type and *A. × proliferum*. The *A. cepa* Aggregatum group (both the PO and SH types) had a higher isorhamnetin-3,4′-diglucoside content compared to the *A. × cornutum* and *A. × proliferum*. The highest isorhamnetin-3,4′-diglucoside content was observed in IPT 237 from the A. cep Aggregatum PO type, while the lowest content was detected in accession IPT211, belonging to *A. × cornutum*.

The quercetin-3-glucoside content did not differ between harvest years, except in the *A. cepa* Aggregatum PO type, where a higher content was observed in the harvest year 2019 compared to harvest year 2018. On average, the *A. cepa* Aggregatum SH type had the highest quercetin-3-glucoside content, while the lowest content was detected in the *A. × cornutum* species. Regarding the individual accessions, the highest quercetin-3-glucoside content was detected in IPT241 (*A. cepa* Aggregatum SH type) and the lowest in IPT 211 (*A. × cornutum*).

The most abundant flavonoid in all shallot accessions was quercetin-4′-glucoside, and it ranged from 287.9 mg/100 g DW in IPT241 (*A. cepa* Aggregatum SH type) to 56.1 mg/100 g DW in IPT210 (*A. × proliferum*). A higher quercetin-4′-glucoside content was observed in the harvest year 2018 in the *A. cepa* Aggregatum SH and PO types compared to the harvest year 2018. The opposite effect was observed for the *A. × cornutum*, while there was no significant difference between harvest years in the *A. × proliferum* species. On average, the highest quercetin-4′-glucoside content was observed in the *A. cepa* Aggregatum SH type, followed by the *A. × cornutum* species, the *A. cepa* Aggregatum PO type and, finally, the *A. × proliferum* species.

The isorhamnetin-4′-glucoside was found to be higher in content in the *A. cepa* Aggregatum SH and PO types in both harvest years compared to the *A. × cornutum* and *A. × proliferum* species, also from both harvest years. The isorhamnetin-4′-glucoside content ranged from 59.2 mg/100 g DW in accession IPT239 (*A. cepa* Aggregatum SH type) to 5.0 mg/100 g DW in accession IPT210 (*A. × proliferum*).

The quercetin content ranged from 36.15 mg/100 g DW in accession IPT241 (*A. cepa* Aggregatum SH type) to 5.16 mg/100 g DW in IPT211 (*A. × cornutum*). On average, the highest quercetin content was observed in the *A. cepa* Aggregatum SH type in the harvest year 2018, followed by the harvest year 2019. There were no significant differences in the quercetin content between other shallot accessions from both harvest years.

The highest flavonoid content was observed in accession IPT241, belonging to the *A. cepa* Aggregatum SH type which, on average, also had the highest flavonoid content in the harvest year 2018, followed by the same type in the harvest year 2019 and the *A. × cornutum* from the harvest year 2019. The lowest flavonoid content was observed in the *A. × proliferum* species.

The highest total antioxidant capacity, measured by both FRAP and DPPH radical scavenging, as well as the total phenolic content, was observed in the *A. cepa* Aggregatum SH type, among which the accessions IPT241 and IPT245 had the highest values. The total antioxidant activity was higher in the harvest year 2018 compared to harvest year 2019. On the other hand, the total phenolic content was higher in the harvest year 2019 compared to the harvest year 2018.

### 3.3. Mineral Content

Individual accessions’ macro- and micro-mineral compositions are shown in Table 3 and Table 4, respectively. On average, the *A. cepa* Aggregatum SH type had the highest macro-element content (Ca, K, P, S and Mg), but the differences between harvest years were significant for all elements except for P, where significant differences between harvest years were observed only for the *A. cepa* Aggregatum SH type (Table 3).

The most abundant macro-element in the shallot bulbs was K and the highest content was observed in the *A. cepa* Aggregatum SH type in the harvest year 2019, while in the harvest year 2018 this type showed comparable K levels to the other tested shallots (Table 3). The Ca content was comparable between harvest years in the *A. × cornutum*, *A. × proliferum* and the *A. cepa* Aggregatum SH type, while in the PO type a higher Ca content was observed in the harvest year 2018 (Table 3). The S content was higher in the harvest year 2019 compared to the harvest year 2018 in all species except in the *A. × proliferum* (Table 3). The Mg content was higher in the harvest year 2019 compared to the harvest year 2018 in all shallots (Table 3).

The *A. cepa* Aggregatum SH type was also the most abundant in the micro-elements B, Cu, Fe, Mn and Zn in both harvest years (Table 4). The Al content did not differ between harvest years or shallot groups, except for the *A. × proliferum*, where the harvest year 2018 was higher in Al compared to the harvest year 2018 (Table 4). Of the shallot species, the most abundant in Li was the *A. cepa* Aggregatum PO type, while both *A. cepa* Aggregatum types were the most abundant in Na (Table 4). The *A. × cornutum* group was the most abundant in Mo content (Table 4).

### 3.4. Discrimination of the Shallot Species and Subtypes Based on a PLS-DA Model

**Figure 1:**
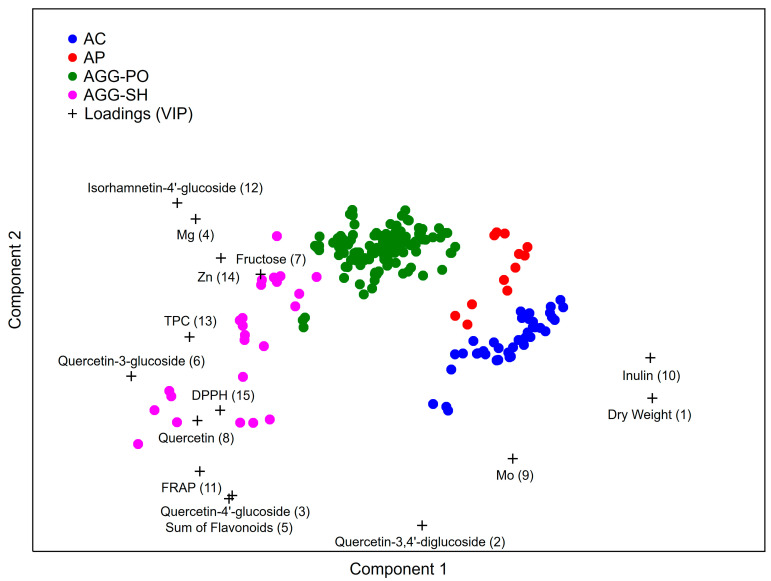
Discrimination of shallot species based on their phytochemical profiles using the PLS-DA model. AC—*A. × cornutum*; AP—*A. × proliferum*; AGG-PO—*A. cepa* Aggregatum potato onion type; AGG-SH—*A. cepa* Aggregatum shallot type.

Based on the 31 analyzed physicochemical parameters, a PLS-DA model was built to discriminate between the investigated shallot species and types, as shown in Figure 1. Among the 31 parameters used to build the model, 15 had a VIP value greater than one and, as such, were considered significant. The most important variables which separated the *A. cepa* Aggregatum types from the *A. × cornutum* and *A. × proliferum* groups were dry weight, fructose, inulin and isorhamnetin-4′-glucoside content. Subsequently, quercetin-3,4′-diglucoside, quercetin-4′-glucoside, the sum of flavonoids, quercetin-3-glucoside and the Zn content were the most important variables in the differentiation between the *A. cepa* Aggregatum SH and PO types, as well as between the *A. × cornutum* and *A. × proliferum* species. Additionally, FRAP, DPPH radical scavenging and TPC were used to distinguish between the *A. cepa* Aggregatum SH and PO types. The Mg and Mo were used to separate the *A. × cornutum* species, while the quercetin content was employed to distinguish the *A. cepa* Aggregatum SH types from the other groups.

## 4. Discussion

The genetic diversity of shallots in Croatia was studied by Puizina [16], with all landraces being determined as belonging to one of the three main species: the *A. cepa* Aggregatum group (2n = 2x = 16), the *A. × proliferum* (Moench) Schrad (2n = 2x = 16) and the *A. × cornutum* Clementi ex Vis. (2n = 3x = 24). In our previous work, we studied 13 shallot landraces from the Croatian coastline for their morphological, biochemical and nutritional diversity [22]. The most important attribute for the shallot species differentiation was flower morphology, but several other morphological, nutritional and biochemical parameters can be used for the discrimination of shallot species [22]. Additionally, two subtypes of *A. cepa* Aggregatum were identified, the *A. cepa* Aggregatum PO type and the *A. cepa* Aggregatum SH type. The *A. cepa* Aggregatum SH type is characterized by an ovate bulb shape and yellow skin color, compared to the broad oval shape and light violet skin color of bulbs of the PO type [17]. In this research, we extended the number of shallot landraces to 34, conducted the experiments over two consecutive harvest years, and included additional biochemical and nutritional parameters to build upon the findings presented in our previous work [22].

### 4.1. Dry Matter, Sugar Profile and Pyruvic Acid Content in the Shallot Accessions

The dry matter content was deemed to be the most important parameter for the differentiation between the *A. cepa* Aggregatum group and species *A. × cornutum* and *A. × proliferum*. The higher dry matter content in the species *A.* × *cornutum* and *A.* × *proliferum* was evident in both harvest years, while there was no difference between the *A. cepa* Aggregatum SH and PO types. The obtained values for the *A. cepa* Aggregatum group’s dry matter content were in line with reported values (between 12.6% and 18.9%) from the literature sources [31,32,33]. Sinclair et al. [34] investigated the dry matter content in 49 *A. cepa* varieties and reported values between 7.4% and 21.5%. The impact of the dry matter on fresh bulb storage is crucial in determining the bulb shelf-life, since higher dry matter content increases the longevity of the product [35].

The most abundant saccharide in shallot bulbs was inulin, with the *A. cepa* Aggregatum accessions having lower inulin content compared to the *A. × cornutum* and *A. × proliferum* accessions. Inulin is a fructan, which consists of the main unit β-(2→1) fructofuranosil and one α-glycopiranose (1→2) terminal unit with a degree of polymerization between 2 to 70, whereas inulin molecules with the degree of polymerization of 2–10 are called fructooligosaccharides [36]. Inulin is recognized as a very important prebiotic [37,38], a non-digestible food constituent that is selectively metabolized by beneficial intestinal bacteria, enhancing their growth and activity. Jaime et al. [39] investigated the sugar profiles of several *A. cepa* varieties and reported a range from 4.02% to 45.8% DW of fructans, 13.7% to 21.4% DW of fructose, 4.7% to 26.38% DW of glucose and 2.7% to 13.6% DW of sucrose. Moongngarm et al. [10] studied the sugar compositions of 13 different foods from Thailand, among which the sugar composition of *A. cepa* Aggregatum was examined, and reported that the bulbs of *A. cepa* Aggregatum contained 33.2% inulin, 6.46% FOS, 11.4% sucrose, 2.91% glucose and 1.57% fructose [10]. The reported values are within the range from our work, except that for sucrose, which they reported with a two-fold higher value. The antioxidant capacity of inulin as a molecule is rather low, as shown by Shang et al. [13]. Nevertheless, there are many studies that show how increased inulin consumption can elevate human plasma antioxidant activity [14,40,41].

Shallots are known and consumed as food and as a condiment due to their pungent flavor. The flavor is produced by the hydrolysis of S-alk(en)yl-L-cysteine sulfoxides and catalyzed by the enzyme alliinase upon tissue damage [42]. Pyruvic acid is a byproduct in this reaction and is widely used as a measure of pungency in Allium species [43,44,45]. The pyruvic acid content varied between harvest years, as well as between shallot species. In fact, we observed an almost two-fold increase from the harvest year 2018 to the harvest year 2019 for all tested shallots except for *A. × proliferum*, where the opposite occurred. On average, *A. × cornutum*, *A. × proliferum* and the *A. cepa* Aggregatum SH type had comparable pungency levels on a dry weight basis. Unlike the dry matter or sugar content, which were comparable among accessions from the same species, the pyruvic acid content range was very broad among the studied accessions and widely overlapped between species. However, if the lower dry matter content is taken into account, we can conclude that both *A. cepa* Aggregatum types are lower in pungency compared to the A. × cornutum and A. × proliferum. Soinien et al. [46] reported on the pyruvic acid content in several edible Allium species, among them the long and round bulb shallots. The authors reported results of 0.14 and 0.36 g/kg fresh weight of pyruvic acid in the long and round shallot bulbs, respectively, which is in line with our findings.

Jadczak et al. [47] studied the effects of different coverings on shallot bulb quality, including the pyruvic acid content. The authors reported a range between 6.30 to 12.25 µmol/g of pyruvic acid per fresh weight over two harvest years, where the content of pyruvic acid doubled in the second harvest year. According to the Schwimmer and Weston [48], with respect to the ranking of onion pungency in our study, the *A. cepa* Aggregatum PO and SH types fall within the extremely mild category (from 2 to 4 µmol pyruvic acid/g fresh weight), whereas the *A. × cornutum* and *A. × proliferum* groups are considered as having mild to intermediate pungency (up to 10 µmol pyruvic acid/g fresh weight).

### 4.2. Antioxidant Activity and Phenolic Compounds Content

Shallots are abundant in phenolic compounds, especially flavonoids, which are responsible for their antioxidant capacity [49]. The obtained values for the TPC and antioxidant capacity of the studied shallot accessions are in line with the values reported in our previous work [22], with several differences. As previously reported, the TPC and antioxidant capacity was higher in *A. × cornutum* and *A. × proliferum* compared to the *A. cepa* Aggregatum group. In our current study, we introduced considerably more shallot accessions, especially from the *A. cepa* Aggregatum group (both PO and SH subgroups), and monitored the TPC and antioxidant capacity over two harvest years. According to our previous work [22], as well as the studies by other authors [7,46,50], the main flavonoid compounds in shallots belong to the flavanol group, among which quercetin-3,4′-diglucoside and quercetin-4′-glucoside are the most abundant. Fredotović et al. [51] studied the bulb flavonoid profile of an *Allium × cornutum* landrace and reported values of 240.01 mg/100 g DW and 159.86 mg/100 g DW for quercetin-3,4′-diglucoside and quercetin-4′-glucoside, respectively. Soininen et al. [46] reported values of 419.8 mg/kg fresh weight and 428.7 mg/kg fresh weight of quercetin-3,4′-diglucoside and 243.3 mg/kg fresh weight and 277.2 mg/kg fresh weight of quercetin-4′-glucoside in round and long bulb shallots, respectively. In our previous work, we reported quercetin-4′-glucoside values ranging from 26.2 to 193.8 mg/kg fresh weight in *A. cepa* Aggregatum bulbs, from 133.5 to 845.0 mg/kg fresh weight in *A. × cornutum* bulbs, and of 213.2 mg/kg fresh weight in *A. × proliferum* [22]. In the present work, we reported values for quercetin-3,4′-diglucoside ranging from 27.0 to 107.0 mg/kg fresh weight in *A. cepa* Aggregatum, from 142.6 to 213.5 mg/kg fresh weight in *A. × cornutum*, and of 124.4 mg/kg fresh weight in *A. × proliferum* [22]. The results obtained in this study are in line with the values reported in our previous work, as well as values reported by the abovementioned authors. Beside the major flavonols, there are several minor quercetin moieties present in the shallot bulbs, such as quercetin-3,7,4′-triglucoside, quercetin-7,4′-diglucoside and quercetin-3-glucoside, as well as isorhamnetin glucosides, such as isorhamnetin-3,4′-diglucoside and isorhamnetin-4′-glucoside [50]. We found that, despite the yearly changes in flavonol content among all the studied shallot accessions, both the *A. cepa* Aggregatum PO and SH types contained significantly more isorhamnetin glucosides based on dry weight compared to the *A. × cornutum* and *A. × proliferum* species. The main difference between the *A. cepa* Aggregatum PO and SH types was the flavonoid content, whereby every flavonoid was more abundant in the SH type compared to the PO type, except for isorhamnetin-3,4′-glucoside. The difference was also obvious in the antioxidant capacity assays, whereby the SH type consistently exhibited higher antioxidant capacity values.

The antioxidant capacities of individual flavonoid molecules have been extensively studied. Zhao et al. [52] showed how isorhamnetin can protect cells from injury by attenuating apoptosis and oxidative stress, while Ganbold et al. [53] studied its hepatoprotective activity. The glycosylated isorhamnetin moieties exhibit an even higher antioxidant activity compared to the aglycone [54]. Quercetin has been shown to have excellent antioxidant properties and has, under specific circumstances, even been deemed comparable in its activity to Trolox [55]. Williamson et al. [56] demonstrated that quercetin glucosides have lower antioxidant and anticancer activity properties compared to the aglycone; however, in the work by Zheng et al. [57], quercetin-4′-glucoside was shown to have the highest antioxidant activity compared to quercetin and its other monoglucosides. Nevertheless, this is another reason why shallots and other Allium species rich in quercetin and its glucosides are considered one of the most health-promoting vegetables and are a ubiquitous part of the Mediterranean diet.

### 4.3. Mineral Content

Averaged over two harvest years, the macro-element (Ca, K, Mg, P, and S) content in accessions belonging to the *A. × cornutum* and *A. × proliferum* groups were largely consistent, while greater diversity was observed in the accessions within both the *A. cepa* Aggregatum SH and PO types. This may be due to the genetic variability in the larger *A. cepa* Aggregatum group compared to the other investigated shallot groups. On the other hand, this variation may be affected by the immobilization of specific minerals in soil, as in the case of Cu, whose uptake by plants depends on the extent of the root’s interception with copper-enriched zones, and the physical properties of the soil, such as the presence of Fe and Mn oxides [58]. Nevertheless, the *A. cepa* Aggregatum SH type had higher concentrations of all macro-elements (Ca, K, Mg, P, and S) as well as several micro-elements (B, Cu, Fe, Mn, and Zn) based on dry weight compared to the other shallots. Building on our previous study [22], we expanded the elemental analysis, encompassing five macro-elements and nine micro-elements in shallots, and the obtained results are within the range of the data published by the USDA [59], as well as with previously published data [60]. From a nutritional standpoint, shallots are abundant in S and K, while at the same time low in Na. They are an excellent source of micronutrients, including Cu, Li, Fe, Mn and Zn. Compared to the common onion, shallots have a five-fold higher Fe concentration and double the amount of Mg, P, K, Zn and Mn [59].

Beside antioxidant activity, such as radical scavenging, flavonoids possess a strong chelating ability for transition metals, such as Cu and Fe. It is assumed that, if the Fe^2+^ ion is still active, free radicals are formed in the immediate vicinity of the metal-flavonoid complex and are scavenged immediately, in which case the flavonoid has a double synergistic action, making it an extremely powerful antioxidant [61]. Among flavonoids, quercetin, which has strong reducing capabilities, has been shown to exhibit a high Fe chelation ability [62]. Furthermore, Kostyuk et al. [63] demonstrated how chelated metal-flavonoid complexes have higher superoxide dismutase activities compared to parent flavonoid molecules, and Porfirio et al. [64] demonstrated that Fe^2+^-quercetin complex has a significantly higher antioxidant activity in the CRAC assay compared to quercetin alone, probably due to the existence of two chelating sites on the quercetin molecule, which promote electron displacement onto the substituents. The combination of the abundance of both the Cu and Fe transition metals, together with high concentrations of quercetin-derived flavonoids, places shallots in a unique position as a potential antioxidant superfood.

## 5. Conclusions

Shallot accessions belonging to the *A. × cornutum* and and *A. × proliferum* groups are characterized by their high dry matter content (around 25%), of which a little less than 50% is formed of inulin-type sugars, polysaccharides, considered an excellent prebiotic with beneficial effects on human health. On the other hand, accessions belonging to the *A. cepa* Aggregatum types have a lower dry matter content and, as a result, lower pungency (measured as the pyruvic acid content), making them more suitable for fresh consumption by a broader range of consumers, but, at the same time, they are abundant in phenolic compounds, especially in quercetin and isorhamnetin glycosides. We also observed greater biodiversity among the accessions within the *A. cepa* Aggregatum group in all the analyzed parameters compared to the other shallot groups. The investigated shallot accessions have an excellent in vitro antioxidant capacity, as well as excellent nutritional properties.

## Figures and Tables

**Table 1:** Shallot dry weight, pyruvic acid content and sugar content by harvest year and species, their interactions and individual accessions.

	Dry Weight	Pyruvic Acid	Inulin	Sucrose	Glucose	Fructose
	%	mmol/kg DW ^1^	g/100 g DW			
**Harvest year**						
2018	19.9 ± 0.5	18.4 ± 0.7	31.7 ± 0.5	1.56 ± 0.07	0.98 ± 0.06	2.79 ± 0.11
2019	16.6 ± 0.6	24.8 ± 0.9	35.1 ± 1.4	4.36 ± 0.16	1.91 ± 0.17	1.57 ± 0.09
*p*-value	***	***	***	***	***	***
**Species**						
*A. × cornutum*	25.4 ± 0.4 a ^2^	22.1 ± 2 ab	47.5 ± 1.1 a	1.64 ± 0.18 c	0.59 ± 0.06 b	1.2 ± 0.07 b
*A. cepa* Aggregatum potato onion type	16 ± 0.2 b	20.4 ± 0.5 c	29.6 ± 0.5 b	3.48 ± 0.17 a	1.73 ± 0.13 a	2.43 ± 0.09 a
*A. cepa* Aggregatum shallot type	14.1 ± 0.8 c	24 ± 2 ab	22.5 ± 1.1 c	3.02 ± 0.29 ab	1.87 ± 0.3 a	3.00 ± 0.36 a
*A. × proliferum*	25.1 ± 0.3 a	27.4 ± 3.5 a	44.8 ± 3.0 a	2.07 ± 0.29 bc	0.64 ± 0.14 b	1.37 ± 0.17 b
*p*-value	***	**	***	***	***	***
**Harvest year × species**						
	***A. × cornutum***					
2018	27.3 ± 0.3 a	16.6 ± 0.5 c	40.7 ± 0.6 b	0.51 ± 0.04 f	0.38 ± 0.06 d	1.42 ± 0.08 cd
2019	23.6 ± 0.2 c	27.6 ± 3.6 ab	54.3 ± 1.8 a	2.77 ± 0.12 c	0.79 ± 0.24 cd	0.99 ± 0.2 d
	***A. cepa* Aggregatum potato onion type**					
2018	17.5 ± 0.2 d	18.1 ± 0.8 c	29.3 ± 0.3 cd	1.9 ± 0.07 de	1.27 ± 0.06 c	2.99 ± 0.1 b
2019	14.5 ± 0.2 e	22.7 ± 0.4 b	29.9 ± 0.8 cd	5.05 ± 0.13 a	2.18 ± 0.06 ab	1.87 ± 0.18 c
	***A. cepa* Aggregatum shallot type**					
2018	16.6 ± 0.1 d	14.8 ± 0.5 c	26.4 ± 0.6 d	1.89 ± 0.03 cde	0.61 ± 0.09 cd	4.63 ± 0.11 a
2019	11.6 ± 0.3 f	33.1 ± 0.8 a	18.7 ± 0.5 e	4.16 ± 0.05 ab	3.13 ± 0.14 a	1.37 ± 0.11 cd
	***A. × proliferum***					
2018	25.6 ± 0.2 ab	35.5 ± 5.3 a	35.2 ± 1 bc	1.12 ± 0.19 f	0.82 ± 0.23 bcd	1.81 ± 0.12 cd
2019	24.5 ± 0.6 bc	19.2 ± 0.2 bc	54.5 ± 1.4 a	3.02 ± 0.32 bcd	0.47 ± 0.31 cd	0.92 ± 0.17 cd
*p*-value	***	***	***	**	***	***
**Accessions**						
	***A. × cornutum***					
IPT021	25.2 ± 0.7 a	39.5 ± 12.2 a	46 ± 4.1 a–d	1.69 ± 0.47 b	0.43 ± 0.07 d	0.8 ± 0.16 c
IPT022	24.9 ± 0.6 a	19.5 ± 2.1 b	47.3 ± 4.2 a–c	1.74 ± 0.4 b	0.81 ± 0.1 d	1.09 ± 0.16 bc
IPT211	26.4 ± 1.2 a	16.3 ± 0.5 b	51.1 ± 3 a	1.54 ± 0.48 b	0.25 ± 0.03 d	0.82 ± 0.23 c
IPT212	26 ± 1.5 a	18.1 ± 0.5 b	46.7 ± 2 a–c	1.76 ± 0.5 b	0.53 ± 0.2 d	1.04 ± 0.12 bc
IPT213	25.8 ± 1.4 a	19.6 ± 0.1 b	46.9 ± 3.4 a–c	1.59 ± 0.54 b	0.74 ± 0.23 d	1.37 ± 0.02 bc
IPT214	25.4 ± 1.6 a	21.9 ± 2.1 ab	45.6 ± 1.7 a–d	1.66 ± 0.6 b	0.63 ± 0.16 d	1.59 ± 0.07 a–c
IPT215	24.5 ± 0.8 a	19.7 ± 0.7 b	48.7 ± 3.1 ab	1.49 ± 0.53 b	0.71 ± 0.18 d	1.71 ± 0.15 a–c
	***A. cepa* Aggregatum potato onion type**					
IPT176	15.5 ± 1.3 bc	27.8 ± 5.3 ab	30.1 ± 2.6 e–i	5.04 ± 0.99 ab	1.41 ± 0.12 b–d	2.3 ± 0.04 a–c
IPT208	15.2 ± 0.1 bc	26.1 ± 3.6 ab	39.2 ± 3.5 a–f	4.6 ± 0.39 ab	1.02 ± 0.04 b–d	2.89 ± 0.27 a–c
IPT216	17 ± 0.9 b	19.3 ± 0.5 b	34 ± 1.4 d–h	4.04 ± 1.11 ab	0.63 ± 0.16 d	1.75 ± 0.43 a–c
IPT217	18.2 ± 1.9 b	20.7 ± 0.3 b	35.1 ± 1.9 c–h	4.31 ± 1.28 ab	1.25 ± 0.18 b–d	2.7 ± 0.08 a–c
IPT218	17 ± 0.5 b	17 ± 1.7 b	37.5 ± 3.2 b–f	3.95 ± 0.91 ab	0.86 ± 0.1 cd	2.41 ± 0.11 a–c
IPT225	16.8 ± 1.3 b	19.6 ± 1.2 b	32.7 ± 0.7 e–i	5.68 ± 1.58 a	0.91 ± 0.07 cd	1.8 ± 0.17 a–c
IPT226	16.4 ± 0.6 bc	20.6 ± 0.4 b	31 ± 1.2 e–i	3.73 ± 0.93 ab	1.29 ± 0.12 b–d	2.39 ± 0.35 a–c
IPT228	14.8 ± 1.7 bc	21.5 ± 2.5 b	26.4 ± 1 g–j	3.64 ± 0.67 ab	2.61 ± 0.75 a–d	2.45 ± 0.09 a–c
IPT229	16.6 ± 0.9 bc	19.8 ± 1 b	30 ± 0.7 f–i	2.55 ± 0.36 ab	2.05 ± 0.23 b–d	3.06 ± 1.05 a–c
IPT230	14.4 ± 1.1 bc	22.1 ± 2 ab	20.9 ± 1.9 ij	4.29 ± 0.95 ab	4.46 ± 0.9 a	2.46 ± 0.29 a–c
IPT231	15.7 ± 1.1 bc	20.9 ± 1.6 b	27.1 ± 0.4 f–j	3.08 ± 0.63 ab	1.71 ± 0.19 b–d	2.57 ± 0.47 a–c
IPT232	16 ± 0.8 bc	19.1 ± 1 b	29 ± 0.2 f–j	2.96 ± 0.48 ab	1.67 ± 0.25 b–d	3.74 ± 0.71 a
IPT233	16.8 ± 0.7 b	18.3 ± 1.9 b	28.4 ± 2.1 f–j	2.8 ± 0.41 ab	2.19 ± 0.95 a–d	1.81 ± 0.2 a–c
IPT234	16.5 ± 0.8 bc	22.2 ± 2.5 ab	27.7 ± 1.9 f–j	2.84 ± 0.57 ab	1.11 ± 0.18 b–d	2.57 ± 0.43 a–c
IPT235	14.9 ± 0.9 bc	16.9 ± 1.6 b	30.1 ± 0.1 e–i	2.92 ± 0.39 ab	1.7 ± 0.23 b–d	2.5 ± 0.25 a–c
IPT236	15.8 ± 1.2 bc	18.4 ± 1.7 b	29.5 ± 0.8 f–j	3 ± 0.56 ab	1.8 ± 0.18 b–d	2.5 ± 0.59 a–c
IPT237	15 ± 0.7 bc	19 ± 2.7 b	26.6 ± 0.5 g–j	2.75 ± 0.52 ab	0.93 ± 0.04 b–d	2.34 ± 0.52 a–c
IPT238	16 ± 1 bc	20.6 ± 3.5 b	26.9 ± 0.9 f–j	2.72 ± 0.63 ab	1.02 ± 0.12 b–d	2.24 ± 0.4 a–c
IPT242	16.3 ± 0.8 bc	22.8 ± 2.8 ab	23.7 ± 1.6 g–j	2.94 ± 0.6 ab	3.28 ± 1.24 ab	2.17 ± 0.18 a–c
IPT243	16.6 ± 1 bc	16.4 ± 1.6 b	27.6 ± 1.1 f–j	2.25 ± 0.38 ab	3.21 ± 0.8 a–c	2.29 ± 0.3 a–c
IPT244	15.3 ± 1 bc	19.8 ± 2.1 b	28.5 ± 0.8 f–j	2.89 ± 0.53 ab	1.15 ± 0.09 b–d	2.06 ± 0.39 a–c
	***A. Cepa* Aggregatum shallot type**					
IPT239	15.1 ± 1.2 bc	22.7 ± 4.3 ab	25.6 ± 1.3 g–j	3.46 ± 0.62 ab	1.12 ± 0.37 b–d	2.86 ± 0.64 a–c
IPT240	15.2 ± 1.2 bc	22.4 ± 3.8 ab	25.8 ± 1.6 g–j	3.48 ± 0.53 ab	1.66 ± 0.56 b–d	3.08 ± 0.41 a–c
IPT241	12.1 ± 2.3 c	26.5 ± 4.8 ab	17.1 ± 2.5 j	3.34 ± 0.65 ab	2.24 ± 0.61 a–d	2.83 ± 0.94 a–c
IPT245	14 ± 1.2 bc	24.2 ± 3.4 ab	21.6 ± 1.7 h–j	1.82 ± 0.27 b	2.44 ± 0.81 a–d	3.23 ± 0.95 ab
	***A. × proliferum***					
IPT023	25.1 ± 0.1 a	21.8 ± 1 ab	42.4 ± 5 a–e	2.26 ± 0.39 ab	1.01 ± 0.18 b–d	1.67 ± 0.27 a–c
IPT210	25 ± 0.6 a	33 ± 6.4 ab	47.2 ± 3.7 a–c	1.87 ± 0.46 b	0.27 ± 0.01 d	1.06 ± 0.14 bc
*p*-value	***	**	***	***	***	**

^1^ DW—Dry Weight; ^2^ Different small letters indicate different homogenous groups using Tukey’s post hoc test. **—*p*-value ≤ 0.01; ***—*p*-value ≤ 0.001.

**Table 2:** Flavonoid compounds, total phenolic content and antioxidant capacity, measured by FRAP and DPPH radical scavenging in shallots by harvest year and species, their interaction and individual accessions.

	Quercetin-7,4′-diglucoside	Quercetin-3,4′-diglucoside	Isorhamnetin-3,4′-diglucoside	Quercetin-3-glucoside	Quercetin-4′-glucoside	Isorhamnetin-4′-glucoside	Quercetin	Sum of Flavonoids	DPPH	FRAP	TPC
	mg/100 g DW ^1^								TEQ µmol/g DW	Fe^2+^ EQ µmol/g DW	GAEQ mg/g DW
**Harvest year**											
2018	1.14 ± 0.05	86.8 ± 3.3	1.96 ± 0.11	8.07 ± 0.46	156.6 ± 5.3	25.7 ± 1.6	8.14 ± 0.69	288 ± 9	9.93 ± 0.26	21.8 ± 0.6	4.42 ± 0.10
2019	1.96 ± 0.04	87.5 ± 2.4	3.09 ± 0.12	8.53 ± 0.43	142.1 ± 4.2	30.2 ± 1.4	12.3 ± 0.95	286 ± 7	6.03 ± 0.22	19.7 ± 0.5	6.09 ± 0.13
*p*-value	***	n.s.	***	n.s.	**	**	***	n.s.	***	***	***
**Species**											
*A. × cornutum*	1.68 ± 0.11 b ^2^	118.4 ± 2.9 a	1.34 ± 0.08 b	4.43 ± 0.3 c	163 ± 4.7 b	10.4 ± 0.7 c	8.12 ± 0.84 b	307 ± 9 b	6.83 ± 0.31 b	20.7 ± 0.6 b	4.63 ± 0.16 c
*A. cepa* Aggregatum Potato onion type	1.37 ± 0.05 c	71.8 ± 1.2 c	2.95 ± 0.11 a	8.01 ± 0.19 b	135.3 ± 2.2 c	33.3 ± 0.9 b	8.12 ± 0.21 b	261 ± 4 c	7.62 ± 0.21 b	19.5 ± 0.3 b	5.17 ± 0.1 b
*A. cepa* Aggregatum Shallot type	1.89 ± 0.1 ab	110 ± 7.6 ab	2.78 ± 0.28 a	17.38 ± 1.13 a	236.7 ± 11.6 a	41 ± 3.9 a	26.63 ± 3.26 a	436 ± 20 a	12.95 ± 0.78 a	30.6 ± 1.3 a	7.36 ± 0.32 a
*A. × proliferum*	2.31 ± 0.2 a	93.6 ± 11.6 b	1.76 ± 0.14 b	6.72 ± 0.69 b	73.8 ± 7.1 d	6.8 ± 0.8 c	6.73 ± 0.36 b	192 ± 20 d	5.79 ± 0.47 b	13.8 ± 0.4 c	4.03 ± 0.22 c
*p*-value	***	***	***	***	***	***	***	***	***	***	***
**Harvest year × species**											
	***A. × cornutum***										
2018	1.08 ± 0.04 c	108.2 ± 4.3 b	0.93 ± 0.08 d	4.59 ± 0.32 d	147.9 ± 5.7 c	7.6 ± 0.6 c	5.7 ± 0.19 c	276 ± 11 c	8.44 ± 0.23	20.1 ± 0.7 cd	3.82 ± 0.1
2019	2.29 ± 0.35 a	128.6 ± 19 a	1.75 ± 0.22 c	4.26 ± 1.11 d	178.2 ± 12.3 b	13.3 ± 1.4 c	10.54 ± 0.51 c	339 ± 35 b	7.17 ± 0.36	21.4 ± 0.8 c	3.37 ± 0.17
	***A. cepa* Aggregatum potato onion type**										
2018	0.93 ± 0.04 c	68.1 ± 2.1 d	2.12 ± 0.11 c	7.27 ± 0.23 c	145.7 ± 3.5 c	29.9 ± 1.3 b	6.69 ± 0.19 c	261 ± 7 c	9.68 ± 0.17	21 ± 0.4 c	4.32 ± 0.07
2019	1.81 ± 0.17 b	75.5 ± 9.5 cd	3.78 ± 0.4 a	8.75 ± 1.49 b	124.9 ± 16 d	36.6 ± 5.8 a	9.56 ± 4.33 c	261 ± 24 c	15.19 ± 0.92	18.1 ± 1.5 de	6.47 ± 0.44
	***A. cepa* Aggregatum shallot type**										
2018	1.82 ± 0.11 b	134.8 ± 2.5 a	3.15 ± 0.07 ab	18.6 ± 0.52 a	266.4 ± 6 a	44.3 ± 1 a	21.06 ± 1.52 b	490 ± 10 a	5.21 ± 0.27	33 ± 1.1 a	5.44 ± 0.18
2019	1.96 ± 0.22 ab	85.1 ± 8.8 c	2.41 ± 0.16 bc	16.17 ± 0.71 a	207.1 ± 6.6 b	37.7 ± 0.7 ab	32.21 ± 0.34 a	383 ± 17 b	4.41 ± 0.25	28.2 ± 0.2 b	4.69 ± 0.06
	***A. × proliferum***										
2018	2.16 ± 0.04 ab	112.8 ± 1.1 ab	1.55 ± 0.1 cd	7.63 ± 0.26 bcd	82.1 ± 2.1 e	6.7 ± 1.1 c	6 ± 0.27 c	219 ± 4 cd	5.56 ± 0.13	13.9 ± 0.3 e	6.02 ± 0.13
2019	2.46 ± 0.12 a	74.3 ± 6.2 cd	1.97 ± 0.39 bcd	5.81 ± 1.69 bcd	65.4 ± 12.3 e	6.9 ± 5.2 c	7.46 ± 4.47 c	164 ± 25 d	10.71 ± 0.88	13.7 ± 1.9 e	8.24 ± 0.29
*p*-value	***	***	***	**	***	*	*	***	n.s.	**	***
**Accessions**											
	***A. × cornutum***										
IPT021	1.69 ± 0.16 ab	127.5 ± 0.8 a–c	1.22 ± 0.18 f–h	4.81 ± 0.21 i–l	157 ± 1.7 d–g	7.8 ± 0.7 ef	6.56 ± 0.34 d	307 ± 2 c–g	7.12 ± 0.82 c–e	18.2 ± 0.6 d–g	3.98 ± 0.24 e
IPT022	1.77 ± 0.25 ab	130 ± 3.8 a–c	1.6 ± 0.09 d–h	4.1 ± 0.8 kl	171.4 ± 7.7 c–f	10.6 ± 0.9 ef	6.08 ± 0.19 d	326 ± 13 c–g	7.07 ± 1.38 c–e	19.7 ± 1.2 d–g	4.26 ± 0.23 de
IPT211	1.62 ± 0.28 ab	107.7 ± 2.1 a–g	1.12 ± 0.09 h	2.5 ± 0.35 l	144.6 ± 3 d–h	7.4 ± 0.7 ef	5.16 ± 0.19 d	270 ± 4 d–g	6.32 ± 0.91 de	18.8 ± 1.5 d–g	4.44 ± 0.2 c–e
IPT212	1.44 ± 0.44 b	112.2 ± 11.1 a–e	1.44 ± 0.33 d–h	5.63 ± 1.69 g–l	165.2 ± 23.4 c–g	12.5 ± 3.1 ef	15.34 ± 4.95 cd	314 ± 45 c–g	7.41 ± 0.27 c–e	21.8 ± 3.2 d–f	4.94 ± 0.73 c–e
IPT213	1.61 ± 0.32 ab	108.7 ± 9.7 a–g	1.18 ± 0.26 gh	4.22 ± 0.44 j–l	153.2 ± 14.9 d–g	8.9 ± 1.5 ef	8.63 ± 1.38 d	286 ± 28 d–g	5.85 ± 0.77 de	21.1 ± 0.5 d–f	4.79 ± 0.49 c–e
IPT214	1.87 ± 0.36 ab	109.8 ± 8.6 a–f	1.45 ± 0.32 d–h	4.81 ± 0.16 i–l	174.4 ± 16.7 c–f	12.6 ± 3.1 ef	7.49 ± 0.7 d	312 ± 30 c–g	6.75 ± 0.58 c–e	23.6 ± 2 c–e	5.07 ± 0.56 c–e
IPT215	1.78 ± 0.29 ab	133 ± 6 ab	1.37 ± 0.14 d–h	4.92 ± 0.51 h–l	175.3 ± 1.5 c–f	13.2 ± 1.1 ef	7.57 ± 0.35 d	337 ± 8 c–f	7.28 ± 0.77 c–e	21.9 ± 0.9 d–f	4.91 ± 0.28 c–e
	***A. cepa* Aggregatum potato onion type**										
IPT176	1.35 ± 0.25 b	68.5 ± 5.2 g–i	2.82 ± 0.32 a–h	6.17 ± 0.5 g–l	133 ± 1.8 d–h	31.1 ± 1.5 b–d	7.46 ± 0.49 d	250 ± 9 f–h	6.01 ± 1.07 de	17.6 ± 0.3 e–g	4.09 ± 0.14 de
IPT208	1.55 ± 0.36 ab	72.3 ± 3 e–i	2.38 ± 0.24 b–h	7.56 ± 0.79 d–k	130.1 ± 8.9 e–h	29.6 ± 1.2 b–d	8.07 ± 0.74 d	251 ± 13 f–h	7.02 ± 1.16 c–e	17.2 ± 1.5 e–g	4.35 ± 0.28 c–e
IPT216	1.38 ± 0.24 b	68.6 ± 5.1 g–i	2.31 ± 0.48 b–h	6.81 ± 0.54 f–k	136.7 ± 2.9 d–h	29.7 ± 3.1 b–d	9.5 ± 1.04 d	255 ± 11 e–g	7.61 ± 0.97 c–e	19.3 ± 1.6 d–g	5.05 ± 0.46 c–e
IPT217	1.36 ± 0.17 b	71.9 ± 2.9 e–i	2.06 ± 0.48 c–h	6.11 ± 0.18 g–l	135.1 ± 8.7 d–h	19.7 ± 3.5 c–e	7.43 ± 0.14 d	244 ± 3 f–h	7.01 ± 1.43 c–e	19.9 ± 1.4 d–g	5.14 ± 0.19 c–e
IPT218	1.41 ± 0.2 b	73.3 ± 3.1 e–i	3.32 ± 0.57 a–e	6.71 ± 0.23 f–l	117.1 ± 6.2 f–i	36.2 ± 0.7 b	6.93 ± 0.67 d	245 ± 8 f–h	7.14 ± 0.83 c–e	17.7 ± 0.8 e–g	4.33 ± 0.21 c–e
IPT225	1.21 ± 0.26 b	71.1 ± 1.5 f–i	3.04 ± 0.71 a–h	6.88 ± 0.82 f–k	105.8 ± 4.2 g–i	31 ± 2.9 b–d	6.63 ± 0.58 d	226 ± 11 gf	7.05 ± 0.63 c–e	17.3 ± 0.6 e–g	4.54 ± 0.55 c–e
IPT226	1.25 ± 0.24 b	72.3 ± 3.6 e–i	3.08 ± 0.76 a–h	9.69 ± 0.22 d–g	135.6 ± 8.2 d–h	31.1 ± 4.1 b–d	7.49 ± 0.46 d	261 ± 5 e–g	7.34 ± 1.37 c–e	19.3 ± 0.5 d–g	5.08 ± 0.43 c–e
IPT228	1.3 ± 0.15 b	73.7 ± 5.7 e–i	2.48 ± 0.35 b–h	8.71 ± 0.32 d–i	138.5 ± 9.2 d–h	33 ± 1 bc	7.19 ± 0.55 d	265 ± 15 d–g	7.74 ± 1.1 c–e	19.8 ± 1.7 d–g	5.22 ± 0.26 c–e
IPT229	1.33 ± 0.21 b	65.1 ± 3.9 i	3.23 ± 0.63 a–f	7.63 ± 0.27 d–k	135.5 ± 3.9 d–h	34 ± 4.4 b	8.73 ± 1.24 d	256 ± 7 e–g	7.52 ± 0.71 c–e	19.5 ± 0.2 d–g	4.88 ± 0.51 c–e
IPT230	1.51 ± 0.35 ab	80.4 ± 6.8 d–i	1.49 ± 0.05 d–h	9.09 ± 1.25 d–h	123.6 ± 5.1 e–h	13.4 ± 2.6 ef	7.94 ± 1.31 d	237 ± 12 f–h	6.98 ± 0.54 c–e	19.1 ± 1.1 d–g	6.46 ± 0.91 a–d
IPT231	1.41 ± 0.35 b	58.5 ± 6.6 i	2.66 ± 0.44 a–h	7.7 ± 0.74 d–k	140.7 ± 1.5 d–h	34 ± 3.5 b	7.73 ± 0.9 d	253 ± 13 e–g	7.21 ± 0.89 c–e	19.6 ± 0.5 d–g	5.2 ± 0.57 c–e
IPT232	1.44 ± 0.18 b	65.2 ± 7.6 i	2.86 ± 0.43 a–h	6.32 ± 0.66 g–l	117 ± 2.3 f–i	29.1 ± 3.9 b–d	6.17 ± 0.36 d	228 ± 15 gh	7 ± 0.47 c–e	18.3 ± 0.4 d–g	4.9 ± 0.33 c–e
IPT233	1.41 ± 0.23 b	75.7 ± 5.6 e–i	3.33 ± 0.38 a–d	8.29 ± 1.3 d–k	129.7 ± 8.3 e–h	34 ± 4.6 b	8.03 ± 1.26 d	260 ± 22 e–g	7 ± 0.68 c–e	18.9 ± 0.7 d–g	5.46 ± 0.51 c–e
IPT234	1.6 ± 0.3 ab	72.7 ± 4.6 e–i	2.93 ± 0.68 a–h	11.21 ± 1.23 de	146.7 ± 2.5 d–h	38.9 ± 4.1 b	9.28 ± 1.08 d	283 ± 10 d–g	8.15 ± 0.88 c–e	21.1 ± 0.5 d–f	5.62 ± 0.54 b–e
IPT235	1.34 ± 0.07 b	73.9 ± 4.2 e–i	3.64 ± 0.11 a–c	6.82 ± 0.37 f–k	126.4 ± 14.6 e–h	36.2 ± 1.8 b	7.42 ± 0.28 d	256 ± 21 e–g	7.64 ± 1.06 c–e	18.4 ± 1.3 d–g	5.34 ± 0.12 c–e
IPT236	1.17 ± 0.15 b	74.9 ± 3 e–i	3.2 ± 0.36 a–g	7.68 ± 0.2 d–k	150 ± 24.3 d–h	36.5 ± 1.6 b	7.17 ± 0.57 d	281 ± 28 d–g	8.85 ± 0.9 b–e	22.5 ± 1.3 de	5.6 ± 0.72 b–e
IPT237	1.62 ± 0.08 ab	98.2 ± 8.9 b–i	4.56 ± 0.19 a	10.65 ± 0.62 d–g	180.2 ± 15.5 c–e	56.7 ± 1.9 a	8.44 ± 0.49 d	360 ± 27 b–e	9.84 ± 1.59 a–e	24.6 ± 2.9 b–e	6.13 ± 0.39 a–e
IPT238	1.36 ± 0.12 b	64 ± 3.9 i	3.03 ± 0.34 a–h	8.74 ± 0.56 d–i	146.5 ± 2 d–h	36.7 ± 1.3 b	8.71 ± 1.11 d	269 ± 6 d–g	8.71 ± 0.93 b–e	20.8 ± 0.9 d–f	5.52 ± 0.48 b–e
IPT242	1.15 ± 0.2 b	70.2 ± 3.9 f–i	2.89 ± 0.24 a–h	9.77 ± 0.32 d–g	152.1 ± 6.2 d–h	34.8 ± 0.9 b	10.9 ± 1.99 cd	282 ± 7 d–g	8.22 ± 0.62 c–e	21.2 ± 0.2 d–f	5.75 ± 0.48 b–e
IPT243	1.12 ± 0.21 b	63.1 ± 4.5 i	2.76 ± 0.37 a–h	7.34 ± 0.19 e–k	122.3 ± 8.2 e–h	31.4 ± 1.2 b–c	8.9 ± 1.08 d	237 ± 4 f–h	7.48 ± 1.04 c–e	18.3 ± 0.7 d–g	4.75 ± 0.32 c–e
IPT244	1.51 ± 0.22 ab	74.8 ± 3.6 e–i	3.82 ± 0.34 a–c	8.37 ± 0.81 d–j	138.9 ± 4.5 d–h	41.3 ± 1.6 b	10.49 ± 0.35 cd	279 ± 2 d–g	8.53 ± 1.13 b–e	19.5 ± 0.5 d–g	5.26 ± 0.33 c–e
	***A. Cepa* Aggregatum shallot type**										
IPT239	1.99 ± 0.1 ab	107.5 ± 6.4 a–h	3.76 ± 0.23 a–c	17.8 ± 0.49 b	248.6 ± 5.2 ab	59.2 ± 2 a	23.2 ± 3.32 bc	462 ± 11 ab	11.87 ± 1.32 a–c	31.2 ± 0.9 ab	6.73 ± 0.44 a–c
IPT240	1.43 ± 0.2 b	91.8 ± 3.5 c–i	4.25 ± 0.14 ab	11.62 ± 0.4 cd	191.3 ± 5.2 b–d	56.3 ± 1.1 a	15.77 ± 2.64 cd	372 ± 9 b–d	10.94 ± 0.67 a–d	25.5 ± 1.3 b–d	6.24 ± 0.53 a–e
IPT241	2.33 ± 0.13 ab	143.2 ± 17.5 a	1.3 ± 0.04 e–h	24.49 ± 0.7 a	287.9 ± 19 a	18.5 ± 1.9 d–f	36.15 ± 9.2 a	514 ± 26 a	15.23 ± 0.13 a	35.7 ± 1.2 a	7.92 ± 0.65 ab
IPT245	1.81 ± 0.21 ab	97.3 ± 19.5 b–i	1.82 ± 0.4 c–h	15.62 ± 2.27 bc	219.1 ± 32.5 bc	29.9 ± 5 b–c	31.41 ± 6.63 ab	397 ± 65 bc	13.76 ± 2.58 ab	30.2 ± 4 a–c	8.53 ± 0.53 a
	***A. × proliferum***										
IPT023	2.79 ± 0.22 a	119.9 ± 17.4 a–d	1.96 ± 0.16 c–h	8.18 ± 1.07 d–k	91.4 ± 9.8 hi	8.6 ± 0.9 ef	7.34 ± 0.3 d	240 ± 29 f–h	6.22 ± 0.8 de	14.4 ± 0.6 fg	4.16 ± 0.2 de
IPT210	1.84 ± 0.2 ab	67.2 ± 2.5 hi	1.57 ± 0.23 d–h	5.26 ± 0.25 h–l	56.1 ± 1.4 i	5.0 ± 0.7 f	6.12 ± 0.59 d	143 ± 3 h	5.36 ± 0.49 d	13.2 ± 0.4 g	3.91 ± 0.4 d
*p*-value	**	***	***	***	***	***	***	***	***	***	***

^1^ DW—dry Weight; *—*p*-value ≤ 0.05; **—*p*-value ≤ 0.01; ***—*p*-value ≤ 0.001. ^2^ Different small letters indicate different homogenous groups using Tukey’s post hoc test. n.s.: not significant.

**Table 3:** Shallot macro-element composition by harvest year and species, their interaction and individual accessions.

	Ca	K	P	S	Mg
	g/kg DW ^1^				
**Harvest year**					
2018	2.16 ± 0.06	10.6 ± 0.3	3.13 ± 0.55	0.91 ± 0.02	0.71 ± 0.01
2019	1.89 ± 0.06	15.8 ± 0.3	3.18 ± 1.14	2.18 ± 0.07	0.88 ± 0.01
*p*-value	***	***	n.s.	***	***
**Species**					
*A. × cornutum*	2.04 ± 0.04 bc ^2^	9.6 ± 0.5 c	2.33 ± 0.26 c	1.25 ± 0.07 c	0.62 ± 0.01 c
*A. cepa* Aggregatum potato onion type	1.87 ± 0.04 c	13.7 ± 0.3 b	3.24 ± 0.54 b	1.53 ± 0.07 b	0.81 ± 0.01 b
*A. cepa* Aggregatum shallot type	2.6 ± 0.22 a	16.6 ± 1 a	4.42 ± 1.48 a	2.23 ± 0.26 a	0.97 ± 0.03 a
*A. × proliferum*	2.41 ± 0.12 ab	12.9 ± 0.8 b	2.62 ± 0.32 c	1.47 ± 0.07 bc	0.86 ± 0.03 b
*p*-value	***	***	***	***	***
**Harvest year × species**					
***A. × cornutum***					
2018	2.16 ± 0.04 a–c	6.7 ± 0.2 e	2.48 ± 0.04 c	0.84 ± 0.02 e	0.54 ± 0.01
2019	1.92 ± 0.07 cd	12.6 ± 0.2 d	2.18 ± 0.05 c	1.66 ± 0.05 c	0.69 ± 0.01
***A. cepa* Aggregatum potato onion type**					
2018	2.08 ± 0.06 bc	11.5 ± 0.2 d	3.25 ± 0.06 b	0.86 ± 0.02 e	0.73 ± 0.01
2019	1.67 ± 0.05 d	16 ± 0.3 b	3.22 ± 0.08 b	2.19 ± 0.06 b	0.89 ± 0.01
***A. cepa* Aggregatum shallot type**					
2018	2.57 ± 0.36 ab	12.6 ± 0.5 cd	3.7 ± 0.17 b	1.05 ± 0.09 de	0.85 ± 0.02
2019	2.64 ± 0.28 a	20.7 ± 1 a	5.15 ± 0.5 a	3.41 ± 0.12 a	1.09 ± 0.04
***A. × proliferum***					
2018	2.22 ± 0.18 a–d	10.3 ± 0.4 d	2.92 ± 0.01 bc	1.42 ± 0.14 cd	0.76 ± 0.03
2019	2.6 ± 0.13 a–c	15.5 ± 0.3 bc	2.32 ± 0.03 c	1.51 ± 0.01 cd	0.96 ± 0.02
*p*-value	*	***	***	***	n.s.
Accessions					
***A. × cornutum***					
IPT021	1.94 ± 0.01 d–j	9.7 ± 1.3 de	2.18 ± 0.04 jk	1.42 ± 0.25	0.63 ± 0.03 f–k
IPT022	2.09 ± 0.01 d–i	10.7 ± 1 c–e	2.39 ± 0.16 h–k	1.22 ± 0.21	0.67 ± 0.01 e–k
IPT211	2.28 ± 0.05 c–g	8.8 ± 1.4 d–e	2.04 ± 0.09 k	1.06 ± 0.12	0.59 ± 0.03 jk
IPT212	2.09 ± 0.07 d–i	10.5 ± 1.8 c–e	2.51 ± 0.03 f–k	1.27 ± 0.15	0.6 ± 0.04 i–k
IPT213	2.00 ± 0.16 d–i	8.5 ± 1.1 e	2.53 ± 0.08 e–k	1.24 ± 0.14	0.63 ± 0.04 g–k
IPT214	1.85 ± 0.08 d–j	9.8 ± 1.5 de	2.43 ± 0.03 g–k	1.39 ± 0.26	0.62 ± 0.05 h–k
IPT215	2.03 ± 0.21 d–i	9.4 ± 1.2 de	2.23 ± 0.06 i–k	1.13 ± 0.16	0.57 ± 0.02 k
***A. cepa* Aggregatum potato onion type**					
IPT176	1.69 ± 0.18 e–j	13.4 ± 0.7 a–e	2.78 ± 0.04 d–k	1.25 ± 0.10	0.84 ± 0.05 a–i
IPT208	3.09 ± 0.27 b	12.1 ± 0.3 b–e	2.77 ± 0.25 d–k	1.13 ± 0.11	0.78 ± 0.03 b–k
IPT216	2.46 ± 0.1 b–d	14.6 ± 1.7 a–e	3.52 ± 0.07 c–g	1.35 ± 0.16	0.89 ± 0.06 a–e
IPT217	1.57 ± 0.16 g–j	10.7 ± 1.5 b–e	3.11 ± 0.26 c–k	1.31 ± 0.15	0.69 ± 0.06 d–k
IPT218	1.59 ± 0.06 f–j	11 ± 0.8 b–e	3.24 ± 0.34 c–j	1.3 ± 0.19	0.66 ± 0.04 e–k
IPT225	1.43 ± 0.01 ij	13.5 ± 1.5 a–e	3.11 ± 0.06 c–k	1.65 ± 0.4	0.72 ± 0.09 d–k
IPT226	1.58 ± 0.14 g–j	14 ± 1.2 a–e	3.73 ± 0.32 b–d	1.47 ± 0.24	0.77 ± 0.05 b–k
IPT228	1.67 ± 0.1 e–j	15 ± 2 a–e	3.61 ± 0.14 c–f	1.9 ± 0.43	0.88 ± 0.05 a–f
IPT229	1.81 ± 0.18 d–j	12.4 ± 1.3 b–e	2.89 ± 0.08 d–k	1.47 ± 0.39	0.76 ± 0.01 c–k
IPT230	1.77 ± 0.06 d–j	12.1 ± 0.4 b–e	3.01 ± 0.01 d–k	1.5 ± 0.41	0.85 ± 0.02 a–h
IPT231	2.3 ± 0.11 c–f	15 ± 1.9 a–e	3.33 ± 0.16 c–i	1.49 ± 0.3	0.86 ± 0.04 a–h
IPT232	1.9 ± 0.12 d–j	12.8 ± 0.8 a–e	2.77 ± 0.03 d–k	1.36 ± 0.21	0.81 ± 0.01 b–k
IPT233	1.72 ± 0.04 e–j	13.9 ± 1.1 a–e	3.21 ± 0.14 c–j	1.75 ± 0.38	0.86 ± 0.03 a–h
IPT234	1.8 ± 0.05 d–j	15.7 ± 0.9 a–d	3.26 ± 0.27 c–j	1.75 ± 0.39	0.84 ± 0.08 a–i
IPT235	2.01 ± 0.02 d–i	14.1 ± 0.6 a–e	2.77 ± 0.11 d–k	1.43 ± 0.22	0.72 ± 0.01 d–k
IPT236	1.8 ± 0.21 d–j	14.2 ± 0.7 a–e	2.85 ± 0.03 d–k	1.58 ± 0.33	0.79 ± 0.05 b–k
IPT237	1.9 ± 0.06 d–j	16.8 ± 1.2 a–c	3.56 ± 0.06 c–f	1.72 ± 0.5	0.83 ± 0.04 b–j
IPT238	1.97 ± 0.13 d–j	15.3 ± 0.8 a–e	3.4 ± 0.05 c–h	1.72 ± 0.53	0.88 ± 0.06 a–g
IPT242	2 ± 0.03 d–j	15.7 ± 1.2 a–d	3.66 ± 0.07 b–e	2.04 ± 0.43	0.98 ± 0.03 a–c
IPT243	1.76 ± 0.17 d–j	11.7 ± 0.4 b–e	3.27 ± 0.13 c–j	1.18 ± 0.2	0.74 ± 0.01 c–k
IPT244	1.53 ± 0.05 h–j	14.4 ± 0.9 a–e	4.14 ± 0.32 bc	1.67 ± 0.27	0.87 ± 0.02 a–g
***A. cepa* Aggregatum shallot type**					
IPT239	2.18 ± 0.07 d–i	13.7 ± 1.3 a–e	2.9 ± 0.08 d–k	2.14 ± 0.7	0.86 ± 0.04 a–h
IPT240	2.96 ± 0.23 b–c	15.6 ± 1.2 a–d	4.15 ± 0.06 bc	2.3 ± 0.52	0.92 ± 0.03 a–d
IPT241	3.99 ± 0.21 a	17.7 ± 2.8 ab	4.75 ± 0.43 b	1.92 ± 0.38	1.08 ± 0.08 a
IPT245	1.29 ± 0.01 j	19.6 ± 2 a	5.89 ± 0.74 a	2.57 ± 0.51	1.02 ± 0.07 ab
*A. × proliferum*					
IPT023	2.37 ± 0.23 c–e	13.2 ± 0.9 a–e	2.6 ± 0.15 e–k	1.32 ± 0.1	0.85 ± 0.07 a–h
IPT210	2.45 ± 0.11 b–d	12.6 ± 1.4 a–e	2.64 ± 0.12 d–k	1.61 ± 0.06	0.86 ± 0.02 a–h
*p*-value	***	***	***	n.s.	***

^1^ DW—dry Weight; *—*p*-value ≤ 0.05; ***—*p*-value ≤ 0.001. ^2^ Different small letters indicate different homogenous groups using Tukey’s post hoc test.

**Table 4:** Shallot micro-element composition by harvest year and species, their interaction and individual accessions.

	Al	B	Cu	Fe	Li	Mn	Mo	Na	Zn
mg/kg DW ^1^									
**Harvest year**									
2018	31.9 ± 0.8	7.98 ± 0.09	6.67 ± 0.12	27.6 ± 0.6	63.3 ± 3.8	10.9 ± 0.2	0.51 ± 0.01	102 ± 4	16.6 ± 0.4
2019	27.7 ± 0.7	6.78 ± 0.09	6.07 ± 0.15	29.6 ± 0.6	42 ± 0.8	10.5 ± 0.1	0.43 ± 0.01	147 ± 5	13.8 ± 0.5
*p*-value	***	***	***	***	***	***	***	***	***
**Species**									
*A. × cornutum*	28.9 ± 0.7	7.86 ± 0.11 b ^2^	5.68 ± 0.19 c	23.9 ± 0.5 c	45.4 ± 1.1 b	10 ± 0.2 c	0.62 ± 0.01 a	83 ± 5 b	11.2 ± 0.3 c
*A. cepa* Aggregatum potato onion type	30.2 ± 0.7	7 ± 0.09 c	6.32 ± 0.1 b	28.8 ± 0.5 b	58.1 ± 3.2 a	10.9 ± 0.2 b	0.42 ± 0.01 c	142 ± 5 a	15.5 ± 0.3 b
*A. cepa* Aggregatum shallot type	29.6 ± 0.9	8.53 ± 0.24 a	8.18 ± 0.32 a	35 ± 1.1 a	47 ± 0.9 ab	11.8 ± 0.2 a	0.49 ± 0.02 b	139 ± 6 a	20.8 ± 1 a
*A. × proliferum*	29.1 ± 3.9	7.33 ± 0.18 bc	5.71 ± 0.17 bc	30.1 ± 1.9 b	32.6 ± 4 b	8.9 ± 0.3 c	0.49 ± 0.03 b	60 ± 4 b	15.1 ± 0.9 b
*p*-value	n.s.	***	***	***	***	***	***	***	***
**Harvest year × species**									
***A. × cornutum***									
2018	29.2 ± 0.8 b	8.54 ± 0.07	6.36 ± 0.25 c	21.4 ± 0.5 e	51.7 ± 1.0	8.8 ± 0.1 e	0.66 ± 0.02 a	63 ± 7	12.3 ± 0.4 cd
2019	28.7 ± 1.2 b	7.19 ± 0.05	5 ± 0.19 d	26.5 ± 0.5 d	39.1 ± 0.6	11.1 ± 0.2 b	0.58 ± 0.01 ab	102 ± 6	10.2 ± 0.3 d
***A. cepa* Aggregatum potato onion type**									
2018	32.1 ± 1 ab	7.61 ± 0.12	6.58 ± 0.15 c	28.1 ± 0.7 cd	72.3 ± 5.9	11.8 ± 0.2 ab	0.48 ± 0.01 c	116 ± 5	17.5 ± 0.4 b
2019	28.3 ± 1 b	6.39 ± 0.09	6.06 ± 0.14 c	29.5 ± 0.7 b–d	43.9 ± 0.6	10.1 ± 0.2 cd	0.36 ± 0.01 d	168 ± 6	13.5 ± 0.4 c
***A. cepa* Aggregatum shallot type**									
2018	32.1 ± 0.9 ab	8.99 ± 0.24	7.92 ± 0.29 ab	32.9 ± 0.7 ab	46.4 ± 0.4	11.2 ± 0.3 a–c	0.48 ± 0.02 c	118 ± 6	18.7 ± 0.6 b
2019	27.2 ± 1 b	8.08 ± 0.38	8.44 ± 0.57 a	37.1 ± 1.9 a	47.6 ± 1.7	12.5 ± 0.3 a	0.51 ± 0.04 bc	159 ± 8	22.9 ± 1.8 a
***A. × proliferum***									
2018	39.9 ± 4.5 a	7.86 ± 0.06	6.26 ± 0.07 b–d	33.8 ± 2.9 a–c	44.3 ± 1.4	8.8 ± 0.5 ed	0.44 ± 0.04 cd	56 ± 3	17.8 ± 0.2 b
2019	18.2 ± 0.4 c	6.81 ± 0.19	5.16 ± 0.05 cd	26.4 ± 1.1 b–e	20.9 ± 3.5	9 ± 0.3 ed	0.54 ± 0.02 bc	64 ± 7	12.3 ± 0.4 cd
*p*-value	***	n.s.	*	**	n.s.	***	***	n.s.	***
**Accessions**									
***A. × cornutum***									
IPT021	27.3 ± 2.6 a–c	7.56 ± 0.33 a–f	4.85 ± 0.32 fg	23 ± 0.2 f–h	40.8 ± 2.1 d	10 ± 0.9 c–g	0.63 ± 0.03 a–d	109 ± 4 d–	9.9 ± 0.2 g
IPT022	32.5 ± 0.9 a–c	7.96 ± 0.35 a–e	5.2 ± 0.42 d–g	27.4 ± 1.3 c–h	43 ± 1.4 d	10.5 ± 0.5 b–g	0.57 ± 0.02 a–g	106 ± 5 d–k	10.6 ± 0.8 fg
IPT211	26.3 ± 0.9 bc	7.76 ± 0.35 a–e	5.06 ± 0.44 e–g	22.8 ± 1.8 f–h	45.4 ± 1.9 d	10 ± 0.5 c–g	0.55 ± 0.02 a–i	59 ± 1 jk	9.7 ± 0.7 g
IPT212	26.2 ± 0.2 bc	8.09 ± 0.21 a–e	5.97 ± 0.19 c–g	24.3 ± 1.8 e–h	47.8 ± 1.8 b–d	10.4 ± 0.7 b–g	0.69 ± 0.01 a	68 ± 11 h–k	11.4 ± 0.3 e–g
IPT213	29.4 ± 1.0 a–c	7.74 ± 0.24 a–e	5.95 ± 0.28 c–g	24.4 ± 0.6 e–h	47.1 ± 3.9 b–d	10.4 ± 0.5 b–g	0.66 ± 0.03 ab	98 ± 27 f–k	12.9 ± 0.5 c–g
IPT214	28.1 ± 0.3 a–c	7.71 ± 0.22 a–e	6 ± 0.25 c–g	22.5 ± 1.2 gh	47.2 ± 4.5 b–d	9.8 ± 0.6 d–g	0.65 ± 0.05 a–c	66 ± 12 i–k	12 ± 0.2 d–g
IPT215	32.7 ± 3.3 a–c	8.23 ± 0.41 a–d	6.75 ± 0.92 b–g	23.3 ± 1.4 f–h	46.5 ± 4.2 cd	8.7 ± 0.1 e–g	0.58 ± 0.01 a–f	75 ± 11 g–k	12.2 ± 1.3 d–g
***A. cepa* Aggregatum potato onion type**									
IPT176	27.1 ± 1.4 bc	6.98 ± 0.35 b–f	6.26 ± 0.25 c–g	25.8 ± 0.5 d–h	43 ± 2 d	12.7 ± 0.4 a–c	0.46 ± 0.03 d–j	242 ± 24 a	13.6 ± 0.7 b–g
IPT208	30.5 ± 2 a–c	7.23 ± 0.71 b–f	4.82 ± 0.26 g	30.2 ± 3.4 b–h	44.9 ± 2.6 d	13.4 ± 0.7 a–c	0.47 ± 0.05 d–j	234 ± 20 ab	15.2 ± 2.6 b–g
IPT216	31.6 ± 0.4 a–c	8.03 ± 0.25 a–e	6.29 ± 0.28 c–g	35.6 ± 0.7 a–c	52.2 ± 3 a–d	12.9 ± 0.6 ab	0.39 ± 0.02 g–j	133 ± 15 c–i	15.7 ± 1.1 b–g
IPT217	31 ± 2.2 a–c	7.28 ± 0.75 a–f	6.87 ± 0.57 b–g	27.9 ± 1.2 b–h	101 ± 28 a–c	11.3 ± 0.4 a–f	0.36 ± 0.02 j	199 ± 1 a–c	15.3 ± 2.4 b–g
IPT218	30.4 ± 0.7 a–c	6.34 ± 0.29 d–f	5.9 ± 0.8 c–g	24.6 ± 0.3 e–h	102 ± 28 ab	9.6 ± 0.5 d–g	0.41 ± 0.04 f–j	143 ± 2 c–g	16.4 ± 3.2 b–g
IPT225	32.4 ± 0.3 a–c	5.66 ± 0.22 f	5.69 ± 0.26 c–g	24.8 ± 2.8 e–h	104 ± 29 a	8.4 ± 0 g	0.45 ± 0.05 d–j	146 ± 20 c–f	15.6 ± 0.8 b–g
IPT226	26.8 ± 0.9 bc	6.53 ± 0.44 c–f	7.65 ± 1.1 a–c	25.5 ± 0.2 d–h	104 ± 29 a	10.2 ± 0.7 b–g	0.38 ± 0.01 h–j	157 ± 9 c–f	19.5 ± 3.1 bc
IPT228	42.6 ± 9.5 a	6.93 ± 0.16 b–f	6.32 ± 0.17 c–g	33.4 ± 2.8 a–e	59.7 ± 13.4 a–d	10.2 ± 0.7 b–g	0.4 ± 0.02 f–j	128 ± 5 d–j	17 ± 0.7 b–f
IPT229	39.8 ± 3.3 ab	6.27 ± 0.32 d–f	5.91 ± 0.26 c–g	30.6 ± 1.1 b–h	43.4 ± 1.3 d	9.8 ± 0.9 d–g	0.4 ± 0.05 f–j	98 ± 12 e–k	12.9 ± 0.7 c–g
IPT230	31.1 ± 5 a–c	7.52 ± 0.11 a–f	6.47 ± 0.19 b–g	34.6 ± 2.3 a–d	45 ± 0.9 d	12.2 ± 0.4 a–d	0.4 ± 0.01 f–j	173 ± 27 a–d	15.8 ± 0.9 b–g
IPT231	24.6 ± 0.6 bc	7.54 ± 0.39 a–f	7.38 ± 0.22 a–d	28.6 ± 1.5 b–h	47.1 ± 0.3 b–d	11 ± 0.4 a–g	0.42 ± 0.03 f–j	110 ± 14 d–k	16.6 ± 0.3 b–g
IPT232	25 ± 1.1 bc	7.09 ± 0.12 b–f	5.62 ± 0.42 c–g	26.4 ± 0.4 c–h	45.7 ± 0.8 d	12 ± 0.9 a–d	0.47 ± 0.1 c–j	123 ± 5 d–j	13.2 ± 1.6 c–g
IPT233	25.9 ± 2.3 bc	6.53 ± 0.21 c–f	5.96 ± 0.11 c–g	27.2 ± 1.4 c–h	45.9 ± 1.1 cd	10.5 ± 0.4 b–g	0.36 ± 0.02 ij	97 ± 12 f–k	14.8 ± 0.6 b–g
IPT234	25 ± 0.9 bc	6.57 ± 0.03 c–f	6.48 ± 0.36 b–g	29.3 ± 2.6 b–h	46.5 ± 1 cd	11.5 ± 0.3 a–e	0.39 ± 0.03 g–j	121 ± 20 d–k	15.5 ± 0.7 b–g
IPT235	25.2 ± 1.5 bc	7.37 ± 0.37 a–f	5.87 ± 0.25 c–g	24.8 ± 0.6 e–h	48.2 ± 0.7 b–d	9.9 ± 0.2 d–g	0.42 ± 0.04 f–j	113 ± 7 d–k	14.2 ± 0.4 b–g
IPT236	24.5 ± 1.9 c	7.09 ± 0.33 b–f	5.31 ± 0.22 d–g	25.9 ± 0.2 d–h	45.8 ± 1 cd	10.8 ± 0.1 a–g	0.43 ± 0.02 e–j	111 ± 8 d–k	12.6 ± 0.8 c–g
IPT237	35.6 ± 3.5 a–c	6.12 ± 0.14 ef	7.12 ± 0.41 b–e	31.7 ± 2.8 a–g	48 ± 0.2 b–d	10.1 ± 0.1 b–g	0.41 ± 0.04 f–j	127 ± 12 d–j	15.8 ± 0.6 b–g
IPT238	38.1 ± 3.4 a–c	6.95 ± 0.58 b–f	7.07 ± 0.42 b–f	34.4 ± 3.6 a–d	46.8 ± 1.1 cd	10.5 ± 0.3 b–g	0.43 ± 0.04 f–j	152 ± 22 c–f	16 ± 0.6 b–g
IPT242	28 ± 2.1 a–c	7.37 ± 0.65 a–f	6.52 ± 0.19 b–g	30.3 ± 0.3 b–h	44.2 ± 0.9 d	11.2 ± 0.6 a–g	0.36 ± 0.01 j	120 ± 11 d–k	17.4 ± 0.5 b–f
IPT243	26.7 ± 0.3 bc	7.9 ± 0.08 a–e	6.23 ± 0.28 c–g	21.8 ± 0.3 h	51.3 ± 1.9 a–d	10.4 ± 0.6 b–g	0.46 ± 0.01 d–j	129 ± 7 c–j	13.8 ± 0.5 b–g
IPT244	32 ± 0.4 a–c	7.72 ± 0.07 a–e	6.98 ± 0.38 b–g	31.6 ± 0.3 a–g	51.6 ± 2.1 a–d	10.8 ± 0.3 a–g	0.49 ± 0.02 b–j	125 ± 2 d–j	18.7 ± 0.8 b–c
***A. cepa* Aggregatum shallot type**									
IPT239	29.2 ± 3 a–c	7.62 ± 0.63 a–f	6.1 ± 0.22 c–g	40.5 ± 2.3 a	45 ± 0.5 d	12 ± 0.8 a–d	0.37 ± 0.01 ij	124 ± 15 d–j	17.8 ± 0.4 b–e
IPT240	29.2 ± 0.3 a–c	8.86 ± 0.55 ab	8.68 ± 0.31 ab	36.9 ± 1.3 ab	45.4 ± 0.2 d	11.2 ± 0.1 a–f	0.42 ± 0.02 f–j	122 ± 7 d–j	20.6 ± 0.3 ab
IPT241	30.3 ± 0.7 a–c	9.24 ± 0.12 a	8.57 ± 0.37 ab	31.1 ± 0.9 b–h	46.5 ± 1 cd	12.8 ± 0.1 ab	0.62 ± 0.04 a–e	169 ± 14 b–e	17.9 ± 0.6 b–e
IPT245	29.9 ± 1.9 a–c	8.4 ± 0.26 a–c	9.38 ± 0.57 a	31.4 ± 0.9 a–g	51 ± 2.8 a–d	11.3 ± 0.3 a–f	0.56 ± 0.02 a–h	139 ± 1 c–h	26.9 ± 2.9 a
***A. × proliferum***									
IPT023	24.5 ± 2.6 c	7.48 ± 0.12 a–f	5.6 ± 0.23 c–g	28.1 ± 0.6 b–h	30.3 ± 7.5 d	8.7 ± 0.5 f–g	0.52 ± 0.02 a–j	71 ± 4 h–k	14.8 ± 1.5 b–g
IPT210	33.6 ± 7.3 a–c	7.19 ± 0.36 b–f	5.82 ± 0.26 c–g	32.1 ± 3.7 a–f	34.8 ± 3.2 d	9.1 ± 0.4 e–g	0.45 ± 0.05 d–j	50 ± 1 k	15.4 ± 1 b–g
*p*-value	***	***	***	***	***	***	***	***	***

^1^ DW—dry Weight; *—*p*-value ≤ 0.05; **—*p*-value ≤ 0.01; ***—*p*-value ≤ 0.001. ^2^ Different small letters indicate different homogenous groups using Tukey’s post hoc test.

## Data Availability

The data are available within this article.
